# A systematic quantitative approach comprehensively defines domain-specific functional pathways linked to *Schizosaccharomyces pombe* heterochromatin regulation

**DOI:** 10.1093/nar/gkae1024

**Published:** 2024-11-20

**Authors:** Abubakar Muhammad, Zsuzsa Sarkadi, Agnisrota Mazumder, Anissia Ait Saada, Thomas van Emden, Matias Capella, Gergely Fekete, Vishnu N Suma Sreechakram, Bassem Al-Sady, Sarah A E Lambert, Balázs Papp, Ramón Ramos Barrales, Sigurd Braun

**Affiliations:** Institute for Genetics, Justus-Liebig-University Giessen, Heinrich-Buff-Ring 17, 35392 Giessen, Germany; BioMedical Center (BMC), Division of Physiological Chemistry, Faculty of Medicine, LMU Munich, Grosshaderner Str. 9, 82152 Planegg-Martinsried, Germany; International Max Planck Research School for Molecular and Cellular Life Sciences, Max Planck Institute of Biochemistry, Am Klopferspitz 18, 82152 Planegg-Martinsried, Germany; BioMedical Center (BMC), Division of Physiological Chemistry, Faculty of Medicine, LMU Munich, Grosshaderner Str. 9, 82152 Planegg-Martinsried, Germany; Synthetic and Systems Biology Unit, Institute of Biochemistry, HUN-REN Biological Research Centre, Temesvári krt. 62, 6726 Szeged, Hungary; HCEMM-BRC Metabolic Systems Biology Lab, Budapesti út 9, 6728 Szeged, Hungary; Institute for Genetics, Justus-Liebig-University Giessen, Heinrich-Buff-Ring 17, 35392 Giessen, Germany; BioMedical Center (BMC), Division of Physiological Chemistry, Faculty of Medicine, LMU Munich, Grosshaderner Str. 9, 82152 Planegg-Martinsried, Germany; Institut Curie, Université PSL, Université Paris-Saclay CNRS UMR3348, 91400 Orsay, France; BioMedical Center (BMC), Division of Physiological Chemistry, Faculty of Medicine, LMU Munich, Grosshaderner Str. 9, 82152 Planegg-Martinsried, Germany; International Max Planck Research School for Molecular and Cellular Life Sciences, Max Planck Institute of Biochemistry, Am Klopferspitz 18, 82152 Planegg-Martinsried, Germany; BioMedical Center (BMC), Division of Physiological Chemistry, Faculty of Medicine, LMU Munich, Grosshaderner Str. 9, 82152 Planegg-Martinsried, Germany; Synthetic and Systems Biology Unit, Institute of Biochemistry, HUN-REN Biological Research Centre, Temesvári krt. 62, 6726 Szeged, Hungary; HCEMM-BRC Metabolic Systems Biology Lab, Budapesti út 9, 6728 Szeged, Hungary; Institute for Genetics, Justus-Liebig-University Giessen, Heinrich-Buff-Ring 17, 35392 Giessen, Germany; BioMedical Center (BMC), Division of Physiological Chemistry, Faculty of Medicine, LMU Munich, Grosshaderner Str. 9, 82152 Planegg-Martinsried, Germany; Department of Microbiology and Immunology, George Williams Hooper Foundation, University of California San Francisco, 513 Parnassus Avenue, San Francisco, CA 94143-0552, USA; Institut Curie, Université PSL, Université Paris-Saclay CNRS UMR3348, 91400 Orsay, France; Synthetic and Systems Biology Unit, Institute of Biochemistry, HUN-REN Biological Research Centre, Temesvári krt. 62, 6726 Szeged, Hungary; HCEMM-BRC Metabolic Systems Biology Lab, Budapesti út 9, 6728 Szeged, Hungary; BioMedical Center (BMC), Division of Physiological Chemistry, Faculty of Medicine, LMU Munich, Grosshaderner Str. 9, 82152 Planegg-Martinsried, Germany; Institute for Genetics, Justus-Liebig-University Giessen, Heinrich-Buff-Ring 17, 35392 Giessen, Germany; BioMedical Center (BMC), Division of Physiological Chemistry, Faculty of Medicine, LMU Munich, Grosshaderner Str. 9, 82152 Planegg-Martinsried, Germany; International Max Planck Research School for Molecular and Cellular Life Sciences, Max Planck Institute of Biochemistry, Am Klopferspitz 18, 82152 Planegg-Martinsried, Germany

## Abstract

Heterochromatin plays a critical role in regulating gene expression and maintaining genome integrity. While structural and enzymatic components have been linked to heterochromatin establishment, a comprehensive view of the underlying pathways at diverse heterochromatin domains remains elusive. Here, we developed a systematic approach to identify factors involved in heterochromatin silencing at pericentromeres, subtelomeres and the silent mating type locus in *Schizosaccharomyces pombe*. Using quantitative measures, iterative genetic screening and domain-specific heterochromatin reporters, we identified 369 mutants with different degrees of reduced or enhanced silencing. As expected, mutations in the core heterochromatin machinery globally decreased silencing. However, most other mutants exhibited distinct qualitative and quantitative profiles that indicate heterochromatin domain-specific functions, as seen for example for metabolic pathways affecting primarily subtelomere silencing. Moreover, similar phenotypic profiles revealed shared functions for subunits within complexes. We further discovered that the uncharacterized protein Dhm2 plays a crucial role in heterochromatin maintenance, affecting the inheritance of H3K9 methylation and the clonal propagation of the repressed state. Additionally, Dhm2 loss resulted in delayed S-phase progression and replication stress. Collectively, our systematic approach unveiled a landscape of domain-specific heterochromatin regulators controlling distinct states and identified Dhm2 as a previously unknown factor linked to heterochromatin inheritance and replication fidelity.

## Graphical Abstract

**Graphical Abstract:**
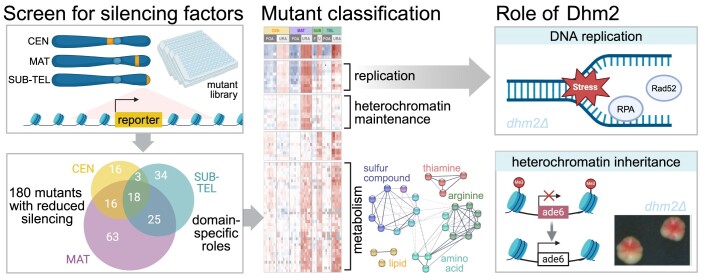

## Introduction

Heterochromatin, a fundamental form of DNA packaging found across eukaryotic genomes, plays pivotal roles in regulating gene expression, maintaining genome stability, shaping chromosomal architecture and determining cell identity. Heterochromatin regions are associated with transcriptionally repressed chromatin and are characterized by a condensed structure, low histone acetylation and the accumulation of specific histone modifications, notably methylation of lysine 9 of histone H3 (H3K9me). This histone mark is recognized by chromodomain proteins and self-propagated through a ‘read-write’ mechanism (1,2). Heterochromatin assembly comprises distinct steps: nucleation, spreading and maintenance. Nucleation involves the DNA- or RNA-guided recruitment of a histone methyltransferase often involving multiple cycles that amplify the initial signal (3). Spreading describes the sequence-independent expansion of H3K9me-marked heterochromatin along the chromosome. Maintenance involves the stable formation of heterochromatin domains and their inheritance during DNA replication. Such inheritance depends on the self-templated propagation of repressive histone modifications as new nucleosomes assemble (1,2). While constitutive heterochromatin persists throughout the cell cycle, often at gene-poor repetitive sequences, facultative heterochromatin forms on specific developmental or lineage-specific genes to stabilize distinct cell states. The spatial positioning of heterochromatin at the nuclear periphery further facilitates its assembly and maintenance (1,4).

The fission yeast, *Schizosaccharomyces pombe*, has distinct constitutive heterochromatin domains present at the pericentromeric repeats, subtelomeres and the silent mating-type locus (5). Many conserved factors in metazoan heterochromatin assembly have orthologs in *S. pombe*, which are encoded by single-copy genes, offering a model system with reduced redundancy and complexity. For example, the homolog of Su(var)3–9, Clr4, is the sole H3K9 methyltransferase in *S. pombe* and catalyzes mono-, di- and trimethylation (6,7). Clr4 associates with a multimeric ubiquitin ligase to form the Clr4-Rik1-Cul4 complex (CLRC) (8–12), which mediates H3K14 ubiquitylation, a prerequisite for H3K9 methylation (13,14). Heterochromatin assembly is initiated by CLRC recruitment to nucleation sites through DNA- and RNA-guided processes. This step involves DNA-binding factors (15–17) and the RNA interference (RNAi) machinery including the argonaute-containing RNA-induced transcriptional silencing complex (RITS) and additional components (18–24). While RNAi is indispensable for heterochromatin establishment and maintenance at pericentromeres, it acts redundantly with DNA-binding factors at subtelomeres and the mating-type locus (15,25). CLRC can also be recruited independently of RNAi to facultative heterochromatin via RNA-elimination factors or components of the telomere-protecting shelterin complex (26–30).

Upon deposition, H3K9me recruits Heterochromatin Protein 1 (HP1) homologs (Swi6 and Chp2) and Clr4 itself, establishing a heterochromatic platform that governs the recruitment of additional heterochromatin factors. Among those factors is the Snf2-like nucleosome remodeler and histone-deacetylase repressor complex SHREC, an ortholog of mammalian NuRD (31–33). HP1 also acts as a docking site for Epe1, a putative H3K9me demethylase that counteracts heterochromatin spreading (34,35). Epe1 distribution on chromatin is confined to the heterochromatin boundaries through selective degradation by the ubiquitin ligase Cul4-Ddb1^Cdt2^, adding another layer of regulation (36). The association of HP1-bound factors is further controlled by phosphorylation and interaction with inner nuclear membrane proteins (37–40). In several systems, heterochromatin assembly is also subject to metabolic regulation and dependent on nutrient availability, such as methionine that serves as a donor precursor for histone methylation (41–43). However, the broader spatio-temporal regulation of heterochromatin and the distinct requirements across different heterochromatin domains remain largely unexplored, underscoring a significant gap in our understanding of heterochromatin biology.

Genetic screens combined with reporter genes that monitor the transcriptional activity in individual heterochromatin domains *in vivo* have emerged as powerful tools for identifying heterochromatin regulators (5). However, previous studies focused on single heterochromatin loci relied on qualitative color-based readouts or semi-quantitative methods with limited resolution. To overcome these limitations, here we adopt a quantitative and systematic approach targeting all major constitutive heterochromatin domains. By quantifying the degree of de-repression in these domains, we systematically determined the requirements for specific heterochromatin regulators. Moreover, by correlating phenotypic profiles across distinct heterochromatin domains, we uncovered striking phenotypic similarities among chromatin regulators belonging to the same complex, suggesting the potential to predict novel functional relationships. Additionally, we identified various metabolic pathway genes specifically required for subtelomeric silencing. Further, we identified and characterized a novel heterochromatin regulator, Dhm2, required for constitutive and facultative heterochromatin maintenance and connected to DNA replication. Our findings yield a substantial body of knowledge that will pave the way for future investigations into heterochromatin regulation.

## Materials and methods

### Yeast strains and media

A modified version of the Bioneer *S. pombe* haploid deletion mutant library (version 3.0), in which non-essential genes were replaced with a *kanMX* cassette, was used for the reporter screens. In this collection encompassing 2988 mutants, several mitochondrial DNA-encoded genes and mutants with severe growth phenotypes had been removed (Supplementary Table S1). All other *S. pombe* strains used in this study are listed in Supplementary Table S14. Gene deletions and yeast strains expressing epitope-tagged proteins were generated by standard genome engineering procedures using transformation with polymerase chain reaction (PCR) products and genomic integration via homologous recombination, as described earlier (44). Generated strains were validated by colony PCR. Reporter strains for monitoring silencing by FC were generated by inserting three transcriptionally encoded fluorescent reporters into the subtelomere of chromosome arm IIR into the SD4 strain from Junko Kanoh’s laboratory (45) using a CRISPR/Cas9-based method (SpEDIT) (46). For the reporters, the gene sequences were codon-optimized for *S. pombe* (47) and the following constructs were used: Superfolder GFP (SF-GFP_s.p._, ‘green’) driven by the *ade6* promoter was integrated proximal to the *tlh2* gene approximately 11 kb downstream of the telomeric repeats; *ade6p*-driven Kusabira orange (mKO2_s.p._, ‘orange’) was integrated at either ∼28 or ∼37 kb from the telomeric repeats; *act1p*-driven E2Crimson (E2C_s.p._, ‘red’) was inserted at the nearest euchromatic region ∼46.5 kb (note: subtelomeric positions are corrected for the ∼4 kb sequence missing at the end of chromosome IIR in the annotated chromosome sequence on www.pombase.org). Reporter strains expressing fluorescent proteins from the mating type locus are derivatives from previously described reporter strains (48). For reverse transcriptase combined with quantitative PCR (RT-qPCR) and chromatin immunoprecipitation (ChIP)-qPCR experiments, cells were grown in rich medium (Yeast Extract Supplemented, YES) at 30°C. For ChIP experiments investigating H3K9me2 and -me3 at the inducible *4xtetO-ade6* locus expressing the *nmt81* promoter-inducible Clr4*-tetR fusion (49), cells were grown in PMG (Pombe Minimal Glutamate) medium. For genetic screens, cells were grown in synthetic medium (Edinburgh Minimal Medium, EMM). EMM medium supplemented with 5-FOA contained 1g/L 5′-fluoroorotic acid.

### Genome-wide screen for heterochromatin factors

Genome-wide screens were performed as described earlier (38,50) with some modifications. Briefly, a haploid deletion mutant library (Bioneer, version 3.0) was crossed with strains harboring the *ura4^+^* reporter gene at different heterochromatic loci by using RoToR HDA colony pinning robot (Singer Instruments). To enable selection of the *ura4 +* reporter under conditions of transcriptional repression, the reporter strains carried an additional hygromycin resistance marker (*hphMX6*) inserted into euchromatin next to the heterochromatin locus, which ensures robust genetic linkage during genetic crosses (Supplementary Figure S1). Following mating, cultures were incubated at 42°C for 4 days to eliminate unmated haploid and non-sporulated diploid cells. Germination was induced by plating spores on YES media containing G418 and hygromycin B. For the readout, cells were transferred onto EMM, EMM lacking uracil (EMM-URA) and EMM supplemented with 5′-fluoroorotic acid (EMM + FOA). Colonies were photographed and sizes were measured by the Gitter R package (https://omarwagih.github.io/gitter/). Relative growth was calculated by dividing colony sizes measured on selective medium (EMM-URA or EMM + FOA) by the respective sizes measured on non-selective medium (EMM). To compensate for plate-dependent variations, all values were normalized to the median values of the individual 384-plates. Log2 values were used for clustering and data visualization. For every reporter, we screened the haploid deletion collection multiple times independently (6–8 biological replicates, except subtelomeres, *n* = 3; each biological replicate contained two technical replicates from the same genetic cross generated by duplication during the germination step).

### Confirmation of gene deletions by PCR or barcode sequencing

Prominent mutants isolated as hits in the screens were tested for the correct deletion. PCR analysis of genomic DNA was used to detect the proper junction of the integrated cassette (*kanMX*) or the absence of the deleted ORF using gene-specific primers. Alternatively, the presence of the strain-specific deletion cassette was confirmed by barcode sequencing. Following purification of genomic DNA and PCR amplification using 5′-GCAGTTTCATTTGATGCTCG-3′ and 5′-TTGCGTTGCGTAGGGGGG-3′ as forward and reverse primers, respectively, the barcode sequence (‘downtag’) was analyzed by sequencing and compared with sequences present in the database (http://pombe.kaist.ac.kr/nbtsupp/ or in Han *et al.* 2010 (51)). Misannotated deletion strains (listed in Supplementary Table S8) were either removed from the analysis or renamed using the correct gene (those mutants are annotated with an asterisk). In some instances, this resulted in two copies of the same mutant in our collection (the original mutant and the corrected mutant, e.g. *swi6* and swi6*); in this case, we kept the explicitly confirmed mutant (i.e. swi6*). Mutants that were contaminated by other mutants (e.g. *clr1*, a subunit of SHREC) or false positives containing a *URA3* harboring plasmid initially used by *Bioneer* to generate the haploid deletion library (e.g. *mst2*, a histone acetylase complex subunit) were excluded from the analysis.

### Threshold settings and statistical methods

To determine the number of silencing and anti-silencing (AS) genes from the screens, we applied the following steps. First, we calculated the mean of the relative growth value from the two technical replicates, which we considered as biological replicate value. Next, to make these values comparable across the four different reporter screens, we scaled the datasets by setting the standard deviation to one and centered them by setting the median of the screens to zero (Supplementary Figure S2). Then, we combined the growth values derived from the two readouts (EMM-URA and EMM + FOA, considering that silencing defects appear as growth inhibition on FOA medium (resulting in negative log_2_ values) and increased growth on medium lacking uracil (positive log_2_ values). Thus, for combining both readouts, we first multiplied the FOA score by (−1) before adding together the two types of scores. We refer to this value as *combined FOA/URA score*. Combining both readouts was necessary, because several strains exhibited strong effects by one readout but weak effects by the other. Next, one-sample Student’s *t*-tests were used to identify mutants whose scores were significantly different from 0 (median of the screens). To define significant hits, we employed a *P*-value < 0.05 and an effect-size threshold for the median combined score of biological replicates > 2.5 (*MAT*, *SUBTEL*, *TEL*) or > 3 (*CEN*; a higher threshold was chosen for *CEN* due to the leaky expression of the *ura4 +* at the *imr1L* locus). The validity of these thresholds was assessed by determining the sensitivity in retrieving known heterochromatin factors (recall) and analyzing the precision of ‘hits’ by examining transcript levels of *ura4 +* directly by RT-qPCR (positive predictive value). To this end, we compiled a list of *bona fide* heterochromatin factors based on available GO functional classification of *S. pombe* genes (GO categories: ‘heterochromatin’ (GO:0000792), ‘chromosome, telomeric region’ (GO:0000781), ‘heterochromatin boundary formation’ (GO:0033696) and a phenotypic term ‘decreased chromatin silencing at subtelomere’ (FYPO:0 004 604)) (Supplementary Table S4). By applying these selection criteria (*P*-value, effect-size threshold), we retrieved recall values of 78%, 67%, 73% and 69% for the *CEN, MAT, SUBTEL* and *TEL* screens, respectively (Supplementary Figure S2 and Supplementary Table S6). Since several *bona fide* factors (e.g. RNAi factors) act redundantly at *MAT, SUBTEL* and *TEL*, they were therefore not detected by these heterochromatin reporters, explaining values of <75%. For determining the precision, we analyzed the transcript levels of *ura4 +* at *CEN*, *MAT* and *TEL* by RT-qPCR for a representative subset of mutants retrieved by the reporter growth-based selection criteria (Supplementary Figure S3). We found an elevated expression level (i.e. >1.5-fold change compared to WT) in 50–92% of the tested mutants (depending on the heterochromatic region; note that *SUBTEL* was not tested; Supplementary Tables S5 and S6).

### Generation of cluster groups

To define heterochromatin domain-specific features of the silencing regulators, we clustered the phenotypic profiles by applying the following steps. First, *k*-means clustering was performed by using the function ‘kmeans’ of the ‘stats’ R package. The number of clusters was estimated by considering the biological functions of the genes. To assess the reproducibility of cluster assignment, multiple rounds of *k*-means clustering (*n* > 10) were performed. In rare cases where genes could be assigned to more than one cluster, cluster assignment was made based on frequency or behavior of related genes (e.g. subunits of protein complexes). Next, hierarchical clustering was performed for each *k*-means cluster by using the function ‘hclust’ of the ‘stats’ R package by calculating Euclidean distance. Both for the *k*-means and the hierarchical clustering, median values of the biological replicates were used, and values of the *SUBTEL* and *TEL* screens were half-weighted. For visualization of the heatmaps, values of the biological replicates (mean values of the technical replicates) were used. Four mutants (out of the 180 hits) were excluded from the *k*-means clustering due to missing values. The R script is available at https://doi.org/10.5281/zenodo.11209900.

### RNA extraction and cDNA synthesis

RNA extraction and complementary DNA (cDNA) synthesis were performed as previously described (36,38). In brief, 50 ml of yeast cells (OD_600_= 0.4–0.8) were centrifuged at 4°C and cell pellets were frozen in liquid nitrogen. Upon thawing on ice, cells were resuspended in 1 ml TRIzol reagent. Following the addition of 250 μl of zirconia/silica beads (BioSpec), cells were lysed by bead beating (Precellys 24, Bertin instruments) for 3 × 30 s with 5 min rest on ice, followed by centrifugation at 13 000 rpm for 15 min at 4°C. Recovered supernatant was extracted twice with chloroform and centrifuged at 13 000 rpm at 4°C for 10 min. Following precipitation with isopropyl alcohol, the pellet was washed twice with 75% EtOH, air-dried and resuspended in 50 μl of RNase free dH2O. RNA concentration and quality were measured using a spectrophotometer (NanoDrop™, Thermo Scientific). Resuspended RNA was treated with DNaseI (Ambion) for 1 h at 37°C. The reaction was stopped by adding 6 μl of inactivation reagent. For cDNA synthesis, 5 μg of DNase-treated RNA was converted into cDNA by reverse transcription using oligo(dT)_20_ primer (50 μM) and 0.25 μl of superscript IV (Invitrogen), according to the manufacturer’s instructions.

### Chromatin immunoprecipitation (ChIP)

ChIP-qPCR was performed as previously described (36,52), with some modifications. Yeast cultures (100 ml) were grown in YES media (or PMG media when expressing the *nmt81* promoter-inducible Clr4*-tetR fusion) to mid-log phase (OD_600_ = 0.6–0.8) at 30°C. Cells were cross-linked by adding formaldehyde to a final concentration of 1% for 20 min at room temperature (RT) by gentle shaking. Cross-linking was stopped by adding glycine to a final concentration of 150 mM for 10 min at RT. Cells were washed twice with 50 ml ice-cold PBS (phosphate-buffered saline) and resuspended in 1 ml lysis buffer A (50 mM HEPES/KOH, pH 7.5, 140 mM NaCl, 1 mM EDTA, 1% Triton X-100 [v/v], 0.1% NaDeoxycholate [w/v]), supplemented with Roche protease inhibitors. Cells were lysed by bead beating (Precellys 24, Bertin instruments) for 6 × 30 s with 5 min rest on ice. Genomic DNA was sheared by sonification (Q800R1 sonicator, QSonica) for 30 min (30-s on/off cycles, 90% amplitude) at 4°C, and cell debris was removed by centrifugation at 14 000 rpm at 4°C for 10 min. Soluble chromatin fractions were incubated with antibodies (anti-H3K9me2, Abcam ab1220; anti-H3K9me3, Active Motif, catalog no 39 161) overnight at 4°C, followed by the addition of 25 μl of Dynabeads ProteinG (Life Technologies). Samples were washed 3× for 5 min at RT with lysis buffer A, 3× with high salt buffer (lysis buffer A containing 500 mM NaCl) and finally with 3× wash buffer (10mM Tris–HCl pH 8.0, 250 mM LiCl,1 mM EDTA, 0.5 mM NP-40 and 0.5% NaDeoxycholate [w/v]) and once with TE buffer (10 mM Tris–HCl pH 7.5, 10 mM EDTA). DNA was de-crosslinked and eluted from the antibodies with ChIP Elution buffer (50 mM Tris–HCl, pH 7.5, 10 mM EDTA, 0.8% sodium dodecyl sulfate [SDS]) at 95°C for 15 min, followed by 65°C for 4 h. Eluted DNA was treated with Proteinase K at 55°C for 30 min and then purified with a ChIP DNA Clean and Concentrator kit (Zymo Research), according to the manufacturer’s instructions.

### Quantitative gene expression (RT-qPCR) and chromatin association (ChIP-qPCR) analysis

RNAs converted to cDNA and immunoprecipitated genomic DNA were quantified by real time PCR using PowerTrack™ SYBR Green Mastermix (Applied Biosystems™) and a QuantStudio 3 or QuantStudio 5 Real-Time PCR instrument (Applied Biosystems™). Primers used for qPCR are listed in Supplementary Table S13. Relative expression values for WT and mutants were calculated by normalizing transcript levels to euchromatin control *act1* and then dividing by the mean of all samples from the same experiment (group normalization), as previously described (52). When analyzing single and double mutants for epistatic interactions, statistical testing was performed using R. Multiple testing was performed using one-way Analysis of Variation (ANOVA) followed by a Tukey’s post-hoc test at a 0.05 significance level.

### Flow cytometry

For gene expression analysis in single cells, flow cytometry (FC) analysis was performed according to a previously described protocol (48). *S. pombe* cells were grown to stationary phase in rich media (YES) and then diluted to a concentration of OD_600_ = 0.1 in YES, followed by incubation at 32°C for 4–5 h prior to FC analysis. The BD Fortessa X-50 instrument (UCSF, San Francisco), equipped with a high-throughput sampler (HTS) module, was employed for flow cytometry analysis. Sample sizes ranged from approximately 2000 to 100 000 cells depending on the growth conditions of the respective strain. Compensation was performed using strains expressing no fluorescent proteins (XFP) and a single-color control XFP (SF-GFPsp, mKO2sp, or E2Csp). Compensated SF-GFP and mKO2 signals were normalized to E2C expressed from a euchromatic control locus in each single cell. Additionally, a maximum expression value for SF-GFP and mKO2 was set based on their expression in a heterochromatin-deficient control strain (*clr4Δ*), where reporters should be in an ON state. However, since the reporters are inserted at heterochromatin domains which are prone to recombination in *clr4Δ* strains, there is a risk of losing these reporters. To overcome this issue, color-negative cells were excluded by setting a minimum cutoff for SF-GFP and mKO2 based on a control strain expressing only E2C that mimics a ‘fully repressed’ state for both reporters. ‘Max’ expression values for SF-GFP and mKO2 were then calculated from these color-positive cells in the no-heterochromatin control strain. Subsequently, SF-GFP and mKO2 signals were scaled to the corresponding ‘max’ values in our analysis strains, and the scaled, normalized signals were plotted in 2D hexbin or density plots for visualization.

For cell cycle progression analysis, cells were fixed in 70% ethanol and then washed twice with 50 mM sodium citrate and incubated with 0.1 μg/μl RNAse A for 2 h at 37°C. The samples were stained with 1 μM Sytox Green nucleic acid stain (Invitrogen, S7020) prior to sonication in a water bath (Diagenode Bioruptor, 2 min and 30 s off/30 s on) and subjected to FC using FortessaX20 (BD Biosciences).

### Live-cell microscopy

Live-cell imaging was essentially performed as described (38). In brief, cells were grown overnight in rich medium (YES) to the logarithmic phase (OD_600_ = 0.4–0.6). Before imaging, cells were attached to lectin (Sigma) coated glass-bottom dishes containing a microwell (MatTek). Cells were imaged using a Zeiss AxioObserver Z1 confocal spinning disk microscope with an EMM-CCD camera (Photometrics, Evolve 512) through a Zeiss Alpha Plan/Apo 100×/1.46 oil DIC M27 objective lens. *Z*-stacks were obtained at focus intervals of 0.4 μm. FiJi/ImageJ software was used to count the number of foci in the yeast cells.

### Color growth/sectoring assay

Cells from red colonies were inoculated at low density in liquid PMG (Pombe Minimal Glutamate) medium and grown at 30°C for 1–2 days to saturation (∼7–8 generations). After dilution in PMG, cells were plated onto PMG agar plates containing low adenine (10 mg/l) at a density of 50–200 cells/plate. Cells were grown for 4–5 days at 30°C to form single colonies; plates were then transferred to 4°C to enhance the formation of red pigment in ade6 OFF cells.

### EdU incorporation

Cells were grown in Edinburgh Minimal Media glutamate (EMMg) (not supplemented with amino acids and bases) until exponential phase. The thymidine analog 5-ethynyl-2'-deoxyuridine (EdU) was added to each culture to a final concentration of 150 μM for 10 min. Cells were fixed in ethanol 70% and washed in PBS and twice in PBS 1% Triton. Cells were resuspended in a freshly made ‘click-it’ solution: 100 mM sodium ascorbate (from freshly made 1 M stock), 2 mM CuSO_4_, 0.5 μM Alexa Fluor 488 azide (invitrogen A10266) in PBS (components were added in this order to PBS). After 1 h incubation on a wheel at RT (in the dark), cells were washed twice in PBS.

### Cell imaging for Ssb3-YFP and EdU incorporation

For live-cell imaging, cells were grown in a filtered EMMg medium. Cells from exponentially growing cultures were centrifuged and resuspended in 1/20 the original volume. A drop of 2 μl was put onto an 8-well slide (Thermo Scientific, ER-201B-CE24) coated with a thin layer of agarose (1.4% in filtered EMMg). Eleven z-stack images (step size of 0.2 μM) were collected using a Nipkow Spinning Disk confocal system (Yokogawa CSU-X1-A1) mounted on a Nikon Eclipse Ti E inverted microscope, equipped with a 100× Apochromat TIRF oil-immersion objective (NA: 1.49) and captured on sCMOS Prime 95B camera (Photometrics) operated through MetaMorph® software (Molecular Devices). For EdU-Alexa-488 and Ssb3-YFP detection, the samples were excited with a 488 nm (Stradus®-Vortran Laser Technology, 150 mW) laser (set at 30% intensity with an exposure time of 100 ms). Images were analyzed with Fiji software and the Z series were converted into 2D projection based on maximum intensity values (for Ssb3-YFP images). Spinning disk image acquisition was performed on the PICT-IBiSA Orsay Imaging facility of Institut Curie.

### Pulse-field gel electrophoresis

Pulsed-field gel electrophoresis (PFGE) was carried out as previously described (53). Quantification was performed using ImageJ. Migrating chromosomes in asynchronous condition and 60 min and 90 min after HU release quantified and normalized to the asynchronous condition.

## Results

### A quantitative and systematic screening approach to identify regulatory factors for all major constitutive heterochromatin domains

To systematically identify factors that regulate constitutive heterochromatin, we used a quantitative reporter assay to screen the *S. pombe* haploid deletion collection for altered heterochromatic silencing. Reporter strains carried the *ura4^+^* gene at the pericentromeres (left innermost repeats of centromere 1, *imr1L; CEN*), the silent mating type locus (downstream of *mat3M; MAT*), the subtelomeres (7 kb downstream of the telomeric repeats of the right arm of chromosome 2; *SUBTEL*) or next to the telomeric repeats (left arm of chromosome 2; *TEL*) (Supplementary Figure S1a) (17,54–56). A hygromycin resistance marker (*hphMX6*) inserted in euchromatin next to the heterochromatin locus allowed selection of the reporter (Supplementary Figure S1b). These reporter strains were crossed with a *kanMX6*-marked collection of 2988 deletion mutants derived from a commercial mutant library of non-essential genes (Bioneer v3) that omits several mitochondrial protein-encoding genes and severely sick mutants (Supplementary Table S1). Large-scale genetic crosses were performed following high-throughput SGA (synthetic gene array) approaches (50,57). To monitor the level of *ura4^+^* silencing after crosses, we quantitatively measured gain or loss of growth on solid media lacking uracil (−URA) and containing 5′-fluoroorotic acid (+FOA), which is converted into a toxic compound by the *ura4^+^* gene product, respectively. We normalized these values to growth on non-selective media (relative growth) to account for pleiotropic effects affecting the overall fitness of the mutants and position effects from neighboring mutants (38). Thus, changes in relative growth are proportional to the degree by which heterochromatin is compromised but independent of any other parameters, allowing the quantitative assessment of changes in heterochromatin states in the individual mutants in a highly reproducible manner (Supplementary Figure S1c).

**Figure 1.**
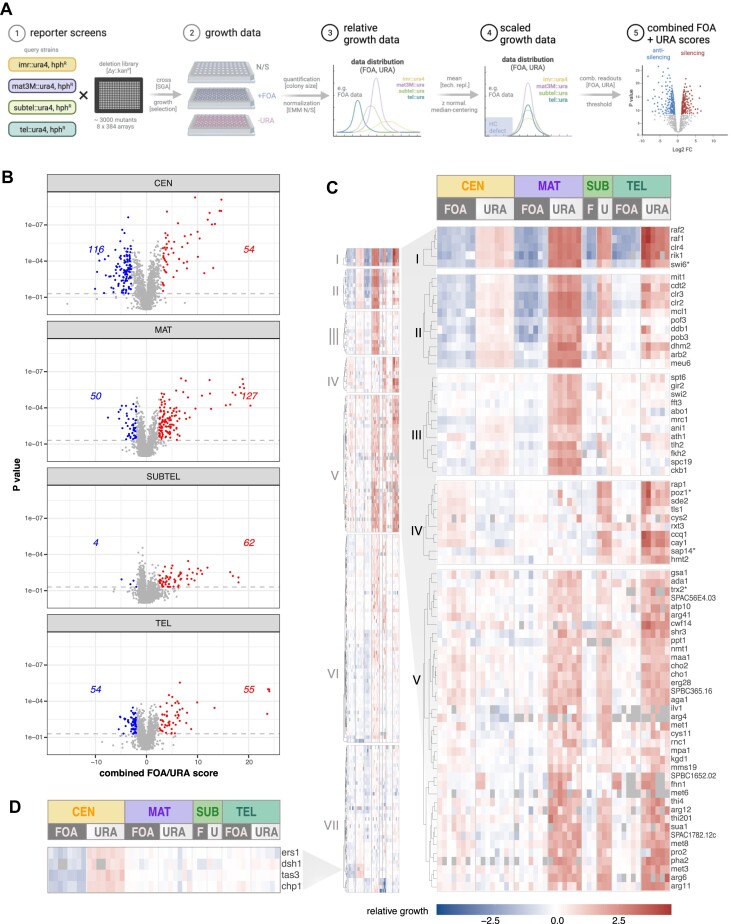
Systematic identification of heterochromatin regulators. (**A**) Schematic overview of the screening strategy employing high-throughput silencing assays. The *ura4^+^* gene in the reporter strains was positioned at the pericentromeric region (*imr1L; CEN*), the silent mating type locus (*mat3M; MAT*), the subtelomeric region (*subtel2R; SUBTEL*) or adjacent to telomeric repeats (*tel2L; TEL*). An additional hygromycin selection (*hphR)* marker was introduced adjacent to the heterochromatic domains. Created in BioRender. Braun, S. (2023) *BioRender.com/y99c398*. (**B**) Volcano plots illustrating the combined FOA/URA scores (*x*-axis) and *P*-values (*y*-axis) derived from one-sample Student’s *t*-test for each mutant (details in ‘Materials and methods’ section). The data include 3–8 independent biological replicates (*CEN*: 8; *MAT*: 7; *SUBTEL*: 3; *TEL*: 6), each comprising 2–4 technical replicates. Mutants with significantly altered silencing (*P*< 0.05) are highlighted and the respective numbers of silencing factors (right side) and anti-silencing factors factors (left side) are indicated. (**C**) Heatmaps displaying *k*-means cluster analysis of log_2_-transformed relative growth values (+FOA, −URA) from 176 silencing mutants exhibiting a significant decrease in heterochromatin silencing in at least one domain (details in the text). The left panel displays an overview of all clusters, while the right panel shows a subset of clusters (I–V) with gene names. The gene order within each cluster was determined through subsequent hierarchical clustering (see ‘Materials and methods’ section). (**D**) Heatmap showing relative growth values of a subset of genes involved in RNAi from Cluster VII. In cases where mutants were mis-annotated in the gene deletion collection, the correct gene name is indicated by an asterisk (see also Supplementary Table S8 and ‘Materials and methods’ section).

For every reporter, we conducted multiple independent screens using the mutant collection (*n* = 3–8; Supplementary Tables S2 and S3) and applied a multistep data processing pipeline to identify mutants significantly affecting heterochromatin silencing (Figure 1A; for details, see ‘Materials and methods’ section). In brief, we applied z-normalization and median-centering to scale the relative growth data, allowing comparison across the four different reporter screens. For each biological replicate, we then combined the normalized values from the two readouts (−URA, +FOA), which we refer to as the *combined FOA/URA score*. This step enhanced the sensitivity of readouts and helped overcome limitations where certain mutants exhibited mild effects in one readout (e.g. +FOA) but substantial effects in the other (e.g. −URA). For high-confidence identification of candidates with altered silencing, we applied a cut-off using a *P*-value < 0.05 and an effect size threshold for the combined +FOA/−URA scores > 2.5 (for *CEN*, we applied a threshold >3 because of the leaky repression of the *imr1L* locus). To assess the validity of these parameters, we evaluated their ability to retrieve known heterochromatin factors employing common GO (Gene Ontology) terms (recall; Supplementary Table S4 and Supplementary Figure S2). In addition, we analyzed the precision of our selection criteria by measuring the transcript levels of the *ura4^+^* reporter gene for a representative subset of mutants (positive predicted value; Supplementary Table S5 and Supplementary Figure S3). Overall, we find a good agreement between reporter growth data and transcript levels (Supplementary Table S6), validating the ability of our growth-based reporter approach to quantitate heterochromatin silencing.

### Identification of factors promoting and antagonizing heterochromatin silencing

By applying these selection criteria, we identified 180 genes that significantly reduced heterochromatic silencing of the *ura4^+^* reporter to various extents. While several mutants affected silencing at multiple heterochromatin domains, the number of hits varied for each reporter locus. The screens performed at *MAT* retrieved the largest number of candidates (127 genes); fewer mutants affected silencing at *CEN* (54), *SUBTEL* (62) and *TEL* (55) (Figure 1B and Supplementary Table S3). We validated the screen by assessing endogenous heterochromatic transcripts by RT-qPCR for a representative selection of mutants. For 42 out of 53 candidates examined (Supplementary Table S7), we detected elevated levels of these transcripts (>1.5-fold relative to WT), largely confirming the growth-based silencing defects in these mutants. The top hits included known members of the core heterochromatin machinery, namely CLRC (Clr4, Rik1, Raf1 and Raf2), SHREC (Clr3, Clr2 and Mit1) and the HP1 homolog Swi6 (Supplementary Figure S4a). These mutants showed highly reproducible values, underscoring the robustness and reproducibility of the quantitative readout and data normalization. In addition, they often displayed reciprocal readouts for the two reporter growth conditions (+FOA, −URA; Supplementary Figure S4a and b). Factors involved in RNAi (Chp1, Tas3, Dsh1, Ers1) were exclusively detected by the *imr1L::ura4* reporter (*CEN*), consistent with their essential function at pericentromeres but redundant role at the other heterochromatin regions. Of note, our screen did not identify certain RNAi components (Ago1, Dcr1, Rdp1, Hrr1) and known heterochromatin factors (Sir2, Chp2, Clr1) due to mis-annotations of the gene deletions or contaminations, as confirmed by genomic PCR and barcode sequencing (Supplementary Table S8; see ‘Materials and methods’ section).

We also identified 189 gene deletions that caused a significant gain in silencing by applying reciprocal selection criteria (effect-size threshold < −2 for *MAT, SUBTEL*, and *TEL* and < −3 for *CEN*). The largest number of factors (116 genes) affected silencing at *CEN*, whereas fewer factors were found to regulate *MAT* (50) and *TEL* (54). Notably, only a few AS factors (4) were identified for *SUBTEL* (Figure 1B). Among factors counteracting silencing, we found components of the RNA polymerase-associated Paf1 complex (Paf1, Leo1), the histone H2B ubiquitin ligase complex HULC (Brl2, Shf1) and the RNA export factor Mlo3 (Supplementary Figure S4c), in agreement with previous reports (50,58–62). Interestingly, lack of the putative H3K9 demethylase Epe1, which prevents heterochromatin spreading beyond its boundaries (34,35), did not significantly affect silencing of any of the reporters in our study. This implies that the absence of Epe1 does not further increase *ura4 +* silencing inserted at those heterochromatin loci, suggesting that intrinsic mechanisms that control Epe1 distribution on chromatin (e.g. Epe1 degradation) are sufficient to prevent its accumulation within heterochromatin in wild-type cells.

In summary, our quantitative and systematic genome-wide screening approach retrieved a large number of factors that positively (180) and negatively (189) control silencing to different extents at constitutive heterochromatin domains.

### A range of factors regulate domain-specific heterochromatin silencing

Beyond the variability in the number of hits identified, we uncovered factors required to silence individual heterochromatin domains. Among the 180 mutants detected, only a small fraction (10 out of 180) affected silencing across all heterochromatin domains, while 26 and 36 impaired three or two regions, respectively (Supplementary Figure S5a and b). In contrast, 108 mutants, more than half, primarily affected a single heterochromatin domain (16 at *CEN*, 67 at *MAT*, 17 at *SUBTEL* and 8 at *TEL*). Only *SUBTEL* and *TEL* exhibited a considerable degree of overlap. Remarkably, when comparing previous genome-wide screens conducted for individual heterochromatin loci (63–68), we noticed similar patterns of limited overlap between hits for different heterochromatin domains (Supplementary Figure S5b–d). However, the number of candidates undisclosed by our study exceeded those of previous studies at the individual scale and even when combined (see ‘Discussion’ section).

To further analyze domain-specific heterochromatin regulation, we performed *k*-means clustering and identified seven distinct clusters, each showing a specific phenotypic profile (Figure 1C and Table 1; Supplementary Figure S6A and Supplementary Table S9). Cluster I displayed silencing defects throughout all heterochromatin regions and contained components mediating H3K9 methylation and spreading (CLRC and Swi6^HP1^). Cluster II exhibits similar defects yet weaker phenotypes for *TEL*. This cluster comprised additional components of the heterochromatin core machinery, including SHREC and the ubiquitin ligase Cul4-Ddb1^Cdt2^ mediating Epe1 degradation within heterochromatin. We also found several factors implicated in DNA replication, consistent with previous reports (Supplementary Table S10) (66,68). Interestingly, while factors promoting RNAi are generally absent in Clusters I and II, the *arb2Δ* mutant differed significantly from other RNAi mutants, displaying a broad silencing defect that was also confirmed by RT-qPCR analysis (Supplementary Figure S7a; see ‘Discussion’ section).

**Table 1.** Clusters and representative mutants with decreased silencing

Cluster	HC domain	Description	Function	Genes	Complex
I	**CEN MAT SUB TEL**	Heterochromatin core machinery	H3K9 methylation	clr4, rik1, raf1, raf2	CLRC
			HC spreading	swi6	HP1
II	**CEN MAT SUB** TEL	Heterochromatin core machinery and DNA replication	HDAC nucleosome remodeling	clr3, clr2, mit1	SHREC
			Ub ligase (E3)	ddb1, cdt2	Cul4-Ddb1^Cdt2^
			DNA replication	pob3 pof3 (F-box) mcl1 (CTF4)	FACT SCF ubi. ligase Polα-associated
III	CEN **MAT**	Heterochromatin maintenance	Nucleosome stability, HC maint. and spreading	fft3, fhk2, abo1, spt6	
			SHREC recruitment	ckb1 mrc1 (Claspin)	Casein kinase II
			Kinetochore assembly	spc19	DASH
IV	**SUB TEL**	Shelterin assembly and maturation	Telomere-end protection	poz1, rap1, ccq1	Shelterin
			Intron-specific splicing	cay1, sde2, tls1	
V	**MAT SUB TEL**	Metabolic processes	Sulfate assimilation	sua1, met1, met3, met8, cys11, mms19 (CIA)	
			Amino acid metabolism	aga1, arg1, arg4, arg6, arg41	
			Thiamine synthesis	nmt1, thi4, thi201	
			Lipid metabolism	cho1, cho2, erg28	
VI	(CEN) MAT SUB	Protein complexes in chromatin organization and broader cellular functions	Histone methylation (H3K4)	set1, ash2, spf1, swd1, swd3	Set1C/COMPASS
			Histone acetyl./deubiquityl.	sgf29, sgf73, spt20	SAGA
			Cohesin-loading/unloading	pds5, wpl1	
			SUMOylation	pmt3, pli1, ulp2, nup132	
			tRNA modification	elp1, elp2, elp3, elp4	Elongator
			Tubulin assembly	pfd3, pfd4, pfd5, pfd6	Prefoldin
VII	**CEN**	other functions	RNAi	chp1, tas3, ers1, dsh1	RITS, RDRC
			Chromatin anchoring	pdp3, ptf1	Mst2C/NuA3

Legend phenotype (HC domain): strong (bold), moderate (regular), weak (brackets)

Cluster III (15 mutants) exhibited a profound defect in *MAT* silencing, while displaying only subtle defects at other regions. Several factors present in this group have been linked to nucleosome stability and heterochromatin maintenance (e.g. the SMARCAD family nucleosome remodeler Fft3 (69)) as well as SHREC recruitment (HP1 phosphorylation by casein kinase II (39)). For others, the underlying mechanisms of silencing remain unclear. Notably, the loss of Spc19, a member of the DASH complex involved in kinetochore assembly, differed markedly from the moderate (or absent) phenotypes observed in other complex members, as confirmed by RT-qPCR (Supplementary Figure S7b). This difference suggests that Spc19 has a unique function independent of its role in DASH.

In contrast, Cluster IV specifically affected *SUBTEL* and *TEL*. This group encompasses members of the telomere-protecting shelterin complex, including Ccq1, Rap1 and Poz1. We also found factors pivotal for the splicing of shelterin components. Notably, silencing in these mutants remained largely unperturbed at *CEN* and *MAT* (Supplementary Figure S7c), corroborating previous findings demonstrating their intron-specific roles in splicing the shelterin components Rap1 and Poz1 (65,70,71). Cluster V was similarly deficient in *SUBTEL* and *TEL* silencing but also displayed defects at *MAT*. Intriguingly, this relatively large group comprised many factors involved in various metabolic pathways (discussed further below).

Clusters VI and VII displayed less severe silencing defects (Supplementary Figure S6a). Cluster VI exhibits defects predominantly at *MAT* and *SUBTEL*. A distinctive feature was the presence of multiple protein complexes involved in chromatin regulation and broader cellular functions, such as Set1C/COMPASS (histone methylation), SAGA (histone acetylation and deubiquitylation), Elongator (tRNA modification), Prefoldin (tubulin assembly) and several factors involved in SUMOylation. While phenotypes in these mutants were subtle, they were highly reproducible and coherent among the complex members. Cluster VII exhibits primarily defects at *CEN* but weaker phenotypes at other regions. Prominent members of this cluster included components of the RNA-induced transcriptional silencing complex (RITS) and RNA-dependent RNA polymerase complex (RDRC), linked to RNAi, and the nuclear membrane protein Dsh1, which tethers these complexes to the nuclear periphery (72). This group also includes two components (Pdp3, Ptf1) of the Mst2C^NuA3^ histone acetyltransferase complex that anchor the complex to H3K36me3-marked euchromatin and prevent its encroachment into heterochromatin (52,61).

Next, we aimed to uncover common characteristics among mutants that enhance silencing. These mutants showed less pronounced effects compared to those that decreased silencing. They further tended to manifest at the *CEN* reporter, consistent with the leaky expression of *ura4^+^* at the *imr1L* locus and its strong repression at other heterochromatic regions in wild-type cells. To categorize the mutant phenotypes, we used k-means clustering, which resulted in seven distinct groups (Table 2; Supplementary Figure S6b and Supplementary Table S9). The AS-Cluster I stood apart as displaying global defects with enhanced silencing at *CEN*, *MAT* and partially at *TEL*. This group encompasses components of nucleosome remodeler Ino80C and factors involved in transcriptional elongation, pre-mRNA 3′-end processing and mRNA export, in agreement with previous findings demonstrating that these processes counteract heterochromatin silencing (50,58–62,73). AS-Cluster II showed enhanced silencing predominantly at *CEN* and comprised the bromodomain protein Bdf2 and H2A.Z-specific histone chaperone SwrC, both preferentially associated with euchromatin (74). SwrC and Ino80 were further shown to prevent heterochromatin spreading (75). In contrast, AS-Cluster III displayed enhanced silencing primarily at *MAT* and comprised the H2B-specific ubiquitin ligase HULC and factors linked to nonsense-mediated mRNA decay. AS-Cluster IV exhibited a similar but weaker pattern and included several factors involved in autophagy.

**Table 2.** Clusters and representative mutants with enhanced silencing

Cluster	HC domain	Description	Function	Genes	Complex
AS-I	**CEN MAT TEL**	Global anti-silencing	Transcriptional elongation	paf1, leo1 tfs1 (TFIIS)	Paf1C
			mRNA 3′-end processing	ctf1	mRNA cleavage/polyadenylation specificity factor complex.
			mRNA export	mlo3	
			Nucleosome remod.	hap2, nht1, iec1	Ino80C
AS-II	**CEN**	Euchromatin-associated	H2A.Z deposition	swr1, swc2, swc3, swc5, msc1, arp6	SwrC
			Acetyl-binding	bdf2	
AS-III	**MAT** TEL	Transcriptional elongation and others	H2B ubiquitylation	shf1, brl2^a^	HULC
			Nonsense-mediated mRNA decay	upf1, upf3	
AS-IV	MAT TEL	Autophagy and other functions	Autophagosome function and assembly	atg2, atg3, atg4, atg2402	
AS-V	**CEN**	Shelterin assembly and maturation	Telomere-end protection	poz1, ccq1	Shelterin
			Shelterin splicing	cay1, sde2, tls1	
AS-VI	**CEN**	Metabolic processes	Sulfate assimilation	sua1, met1, met8	
			Amino acid metabolism	aga1, arg4	
AS-VII	**CEN**	diverse cellular functions	Ub-depend. proteolysis	dss1	Proteasome (lid)

**Legend** phenotype (HC domain): **strong (bold)**, moderate (regular)

^a^Note: Data for *brl2Δ* were not available for SUBTEL; therefore, this mutant was not included in the *k*-means cluster analysis. However, the *blr2* mutant showed a similar behavior as *shf1*Δ at other heterochromatin domains.

Intriguingly, several mutants with enhanced silencing at *CEN* displayed the opposite phenotype at *SUBTEL* and *TEL* (i.e. decreased silencing) and were therefore identified by both strategies. This was particularly evident for genes controlling shelterin composition and assembly (Silencing Cluster III, AS-Cluster V; Supplementary Figure S6a and b). These factors do not generally antagonize heterochromatin but rather have a context-specific role, consistent with previous reports (38,76). Interestingly, we observed a similar trend for several mutants affecting metabolic pathways (Silencing Cluster V, AS-Cluster VI).

In conclusion, beyond identifying novel factors, the sensitivity of our quantitative screening approach and the generation of phenotypic profiles across different chromatin contexts let us assign factors to distinct pathways with heterochromatin region-specific functions.

### Phenotypic profiles reflect the submodular architecture of chromatin organization complexes

**Figure 2.**
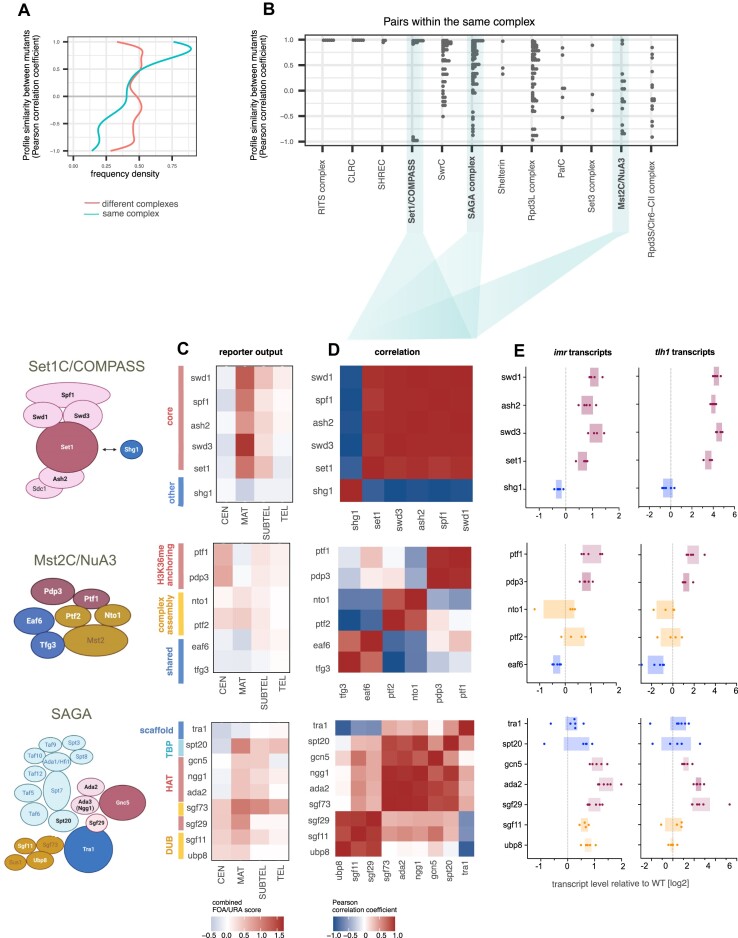
Similarities between phenotypic profiles reflect the composition of protein complexes involved in chromatin organization. (**A**) Density plot showing frequency density of Pearson correlation coefficients for the pairwise correlation of mutant profiles of 70 genes belonging to 12 complexes involved in chromatin organization (for details, see Supplementary Table S11 and the text). Lines represent gene pairs within the same or different complexes (legend is shown below graph). (**B**) Detailed view of pairwise correlations restricted to genes within identical complexes. Pearson correlation coefficients in (A) and (B) were calculated using average values of the combined FOA/URA score from 3 to 8 biological replicates. (**C**) Heatmaps showing the median log_2_-transformed values of the combined FOA/URA scores from 3 to 8 biological replicates (*CEN*: 8; *MAT*: 7; *SUBTEL*: 3; *TEL*: 6; each with two technical replicates) focusing on three complexes: Set1C/COMPASS (top), Mst2C^NuA3^ (middle) and SAGA complexes (bottom). Accompanying diagrams (left panels) depict the complex structures based on existing crystallographic data or functional genetics studies. (**D**) Correlation matrices highlighting Pearson correlation coefficients based on combined FOA/URA scores among subunits within each complex. (**E**) Expression analysis of endogenous transcripts at two heterochromatic loci (*imr1L* and *tlh1*) analyzed by RT-qPCR in selected mutants. Transcript levels are normalized against *act1* levels and presented as box plots using log_2_-transformed values relative to the wild-type (WT) median across biological replicates (*n* = 4–5). Colors correspond to the different protein complex modules.

We noted strikingly similar phenotypic profiles among several components of physical protein complexes, such as CLRC, SHREC and RITS (Figure 1C and D). This observation prompted us to systematically explore whether genetic perturbations in complexes associated with specific chromatin functions typically exhibit distinct phenotypic profiles. To this end, we calculated pairwise similarities between phenotypic profiles among 70 genes linked to 12 established chromatin organization complexes (Supplementary Table S11). Notably, genes encoding subunits of the same complex displayed higher similarities compared to those of different complexes (*P*< 10^−4^ from permutation test, see ‘Materials and methods’ section; Figure 2A). Specifically, 24.1% of within-complex pairs show a Pearson correlation coefficient of at least 0.9, whereas only 7.2% of between-complex pairs reached this level of correlation. This high level of correlation was particularly seen for members of the CLRC, SHREC, RITS and shelterin complexes. However, we noticed that several larger protein complexes, such as Set1C/COMPASS, SAGA and Mst2C^NuA3^, displayed greater diversity, with two or more gene correlation clusters within the same complex (Figure 2B). Focusing on these chromatin complexes, we investigated whether the mutant profiles align with the modular architecture of these complexes.

Based on the reporter growth data (Figure 2C and Supplementary Figure S8), correlation matrices were generated for each complex (Figure 2D). For the Set1/COMPASS histone H3K4 methyltransferase complex (77), the loss of its core subunits (Set1, Spf1, Swd1, Swd3, Ash2) resulted in highly similar phenotypic profiles (Figure 2D, top). Notably, these profiles markedly differed from that of Shg1, a protein that is dispensable for H3K4 methylation and only peripherally associated with Set1C (78). A similar pattern was observed when examining endogenous heterochromatic transcripts by RT-qPCR, revealing an increase in core subunit mutants while no change was seen in cells lacking Shg1 (Figure 2E, top). This indicates that Shg1 is dispensable for heterochromatin regulation. Similarly, distinct phenotypic profiles were identified among subunits of the Mst2C/NuA3 acetyltransferase complex, responsible for modifying histone H3K14 and other chromatin-associated proteins (Figure 2D, middle) (52,61,79). Although the structure of this HAT complex remains elusive, correlating these phenotypic profiles revealed specific clusters (Figure 2D middle), which were also reflected by changes in heterochromatic transcript levels (Figure 2E, middle). Notably, these clusters corresponded with the roles of individual subunits in various functions: complex assembly (Nto1, Ptf1), anchoring of Mst2C to H3K36me3-marked chromatin (Pdp3, Ptf1) and shared activities with other chromatin complexes (Eaf, Tfg3), suggesting a modular architecture for Mst2C. Finally, when examining the multi-functional transcriptional co-activator complex SAGA (77,80), phenotypic profiles of the individual also segregated into the functional modules, including HAT (histone acetylation), DUB (histone deubiquitylation) and the TF-binding module (Figure 2D,E, bottom). However, some noticeable deviations were also observed. The phenotypic profile of *sgf73*Δ differed significantly from other DUB subunits, causing stronger silencing defects. This finding is consistent with the additional role of Sgf73 in RITS assembly independent of its function inside the SAGA complex (81). Furthermore, we found that the phenotypic profile of Sgf29, which is part of the HAT module, correlated with components in the DUB module, suggesting additional roles of Sgf29 within SAGA.

In conclusion, our reporter-based phenotypic profiles not only offer crucial information about the roles of chromatin complexes but also provide insights into their submodular architecture and the functions of these modules in relation to silencing.

### Metabolic pathway genes regulate subtelomeric and telomeric silencing

As mentioned earlier, mutants in Cluster V exhibited a distinct profile with reduced silencing defects at *MAT*, *SUBTEL* and *TEL*, while silencing at *CEN* was enhanced (Figure 1C and Supplementary Figure S6a and b). To obtain insights into the roles of these genes, we conducted a GO term analysis using the AnGeLi web-based tool (82). The analysis revealed significant enrichments in various, partially overlapping metabolic processes, including amino acid metabolic processes (GO: 0006520), arginine biosynthetic processes (GO:0006526), sulfur compound metabolic processes (GO:0006790) and thiamine metabolic processes (GO:0006772) (Supplementary Figure S9a and Supplementary Table S12).

**Figure 3.**
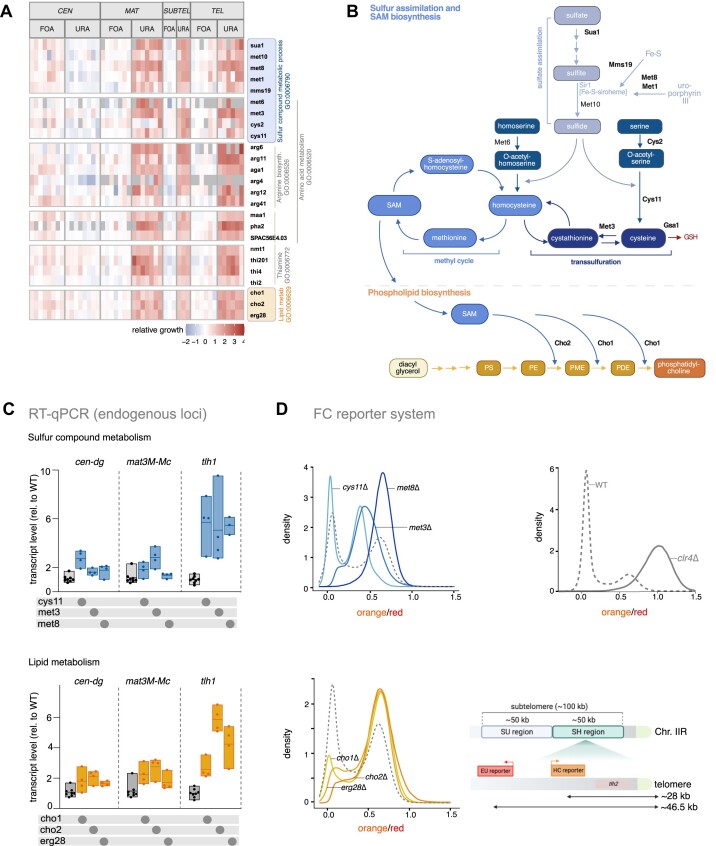
Metabolic pathway genes contribute to silencing at subtelomeres and mating type locus. (**A**) Heatmap depicting relative growth values (+FOA, −URA) of mutants specifically impaired in silencing at *MAT*, *SUBTEL* and *TEL*. Gene order reflects functional groups identified by GO terms. (**B**) Schematic representations of sulfate assimilation (top) and phosphatidylcholine synthesis (bottom) metabolic pathways. Bold protein names correspond to mutants identified under stringent selection criteria. Created in BioRender. Braun, S. (2023) *BioRender.com/x78c746*. (**C**) Quantification of heterochromatin transcript levels at *cen-dg* (pericentromeres), *mat3M::ura4* (mating type locus) and *tlh1* (subtelomeres) by RT-qPCR. Transcript levels from 3 to 4 biological replicates, normalized against *act1* levels, are presented relative to the wild-type (WT) median (*n* = 8). Individual replicates are illustrated in a floating bar plot with the median indicated by a line. (**D**) Density plots displaying FC analysis of fluorescent mKO2 reporter expression at the single-cell level from a subtelomeric locus (HSS^Subtel^ reporter system). FC experiments were conducted in mutants impaired in methionine and cysteine synthesis (top) and membrane lipid synthesis (controls: WT, dashed line; *clr4*Δ, solid line). The *x*-axis shows mKO2 reporter expression values (‘orange’) normalized against E2C expressed from a proximal euchromatic locus (noise filter, ‘red’). The *y*-axis represents the density of the cell population relative to the mean expression value in *clr4*Δ (ON state). The bottom right scheme illustrates the *HSS^Subtel^* reporter system with ‘orange’ and ‘red’ inserted at ∼28 and ∼46 kb, respectively, downstream of telomeric repeats on chromosome IIR.

Examining these genes and their interactions using the STRING database (https://string-db.org/; (83), we identified several genes (*sua1, met1, met3, met6, met8, cys11, mms19*) involved in distinct steps of sulfur assimilation and the biosynthesis of homocysteine, methionine, cysteine and S-adenosyl-methionine (SAM) (Figure 3A and B and Supplementary Figure S9b–d). SAM serves as a universal methyl donor for methylation reactions and may be specifically limiting for H3K9 methylation in these mutants. Additionally, we identified two genes (*cho1, cho2*) encoding SAM-dependent methyltransferases involved in the last steps of phosphatidylcholine synthesis, suggesting the potential importance of membrane composition in the silencing of these heterochromatin domains (Figure 3A and B). Supporting this idea, cells lacking the endoplasmic reticulum (ER) protein Erg28, which tethers several enzymes involved in ergosterol synthesis to the ER membrane, exhibited a similar phenotypic profile (Figure 3A and Supplementary Figure S9d). Other genes in this cluster contribute to thiamine (*nmt1, thi4, thi201*) and arginine synthesis (*aga1, arg4, arg6, arg11, arg12, arg41*), or are generally involved in amino acid metabolism (*maa1, pha2, SPAC56E4.03*) (Supplementary Figure S9d). We also noted that three additional mutants associated with these pathways (*met10, cys2, thi2*) displayed similar phenotypic profiles (Figure 3A), although they were initially not identified as candidates due to our stringent selection criteria (as described above). Collectively, the shared distinct phenotypic profiles of these mutants suggest that these genes play specific roles in the silencing of subtelomeric heterochromatin and the mating type locus. This notion is further supported by the finding that genes from these metabolic pathways, while significantly enriched in Cluster V, are mostly absent in the other groups, as determined by Fisher’s exact test (Supplementary Figure S9e).

Given the prominent roles of genes involved in SAM production and phospholipid biosynthesis, we focused on selected mutants and validated the silencing defects by examining endogenous transcripts from different heterochromatin regions (Figure 3C). Consistent with the growth-based reporter assays, RT-qPCR analysis revealed a moderate but reproducible increase (three to sixfold) in the subtelomeric *tlh1* transcript level in mutants deficient in sulfur assimilation (*cys11, met3, met8*) and lipid metabolism (*cho1, cho2, erg28*). Other heterochromatic transcripts (*cen-dg, mat-Mc*) were either less affected or unaffected, confirming that defects are chromatin context-dependent in these mutants.

To explore if bulk assays mask cell-to-cell differences in heterochromatin behavior in these mutants, we devised a fluorescent reporter system for measuring heterochromatin silencing in individual cells via FC, as described previously (48). In this approach, we integrated a reporter gene encoding Kusabira Orange (‘orange’) 28 kb upstream of the telomeric repeats (Chr2R, Figure 3D). This specific locus exhibits lower levels of repression and is notably more sensitive to chromatin perturbations in comparison to other subtelomeric loci (A. Mazumder, B.A.-S. and S.B., unpublished results). To normalize signals from ‘orange,’ we integrated an additional reporter, E2Crimson (‘red’), in nearby euchromatin. In a wild-type background, the reporter strain exhibited a bimodal behavior between a fully repressed (OFF) and an intermediately de-repressed (ON) state, whereas it was completely de-repressed in cells lacking the methyltransferase Clr4. In the context of this reporter strain, mutants deficient in sulfate assimilation/homocysteine synthesis (*met3, met8*) displayed a pronounced shift towards the intermediate ON state, whereas cells deficient in cysteine synthesis (*cys11*) were less affected (Figure 3D, left top panel). Notably, lipid biosynthesis mutants (*cho1, cho2, erg28*) displayed an even stronger shift to the intermediate ON state (Figure 3D, left bottom panel). In addition to redistributing cells between ON and OFF states, some mutants additionally populate new intermediate ON states not found in wild-type (*cys11* and *met3*). Therefore, together it stands to reason that homocysteine and phospholipid biosynthesis pathways are central to determining gene expression minima and maxima of bimodally distributed heterochromatin loci.

In summary, our phenotypic profiles uncover distinct and unanticipated roles for diverse metabolic pathways in the regulation of heterochromatin at subtelomeric domains. These regions appear to be more susceptible to cellular perturbations than other chromosomal regions.

### Dhm2 is a novel factor involved in silencing at constitutive and facultative heterochromatin

**Figure 4.**
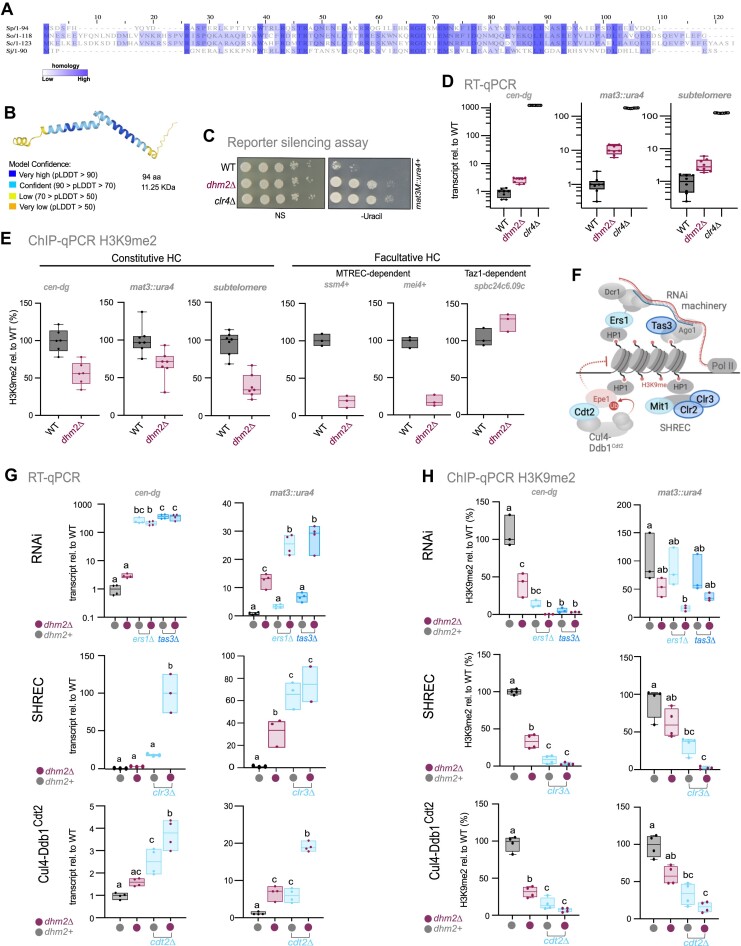
Dhm2 contributes to heterochromatin structure and silencing at constitutive and facultative heterochromatin. (**A**) Multiple sequence alignment of the Dhm2 protein sequence and its homologs present in *Schizosaccharomyces osmophilus*, *Schizosaccharomyces cryophilus* and *Schizosaccharomyces japonicus* using the T-coffee alignment tool. Conservation levels are highlighted (dark shading indicates high conservation). (**B**) Predicted Dhm2 structure based on the AlphaFold2 model. (**C**) Silencing assay using the *mat3M::ura4^+^* reporter. Ten-fold dilutions of wild-type (WT) cells, *dhm2Δ* and *clr4Δ* strains were plated on non-selective (N/S) and selective (lacking uracil) media. (**D**) Quantification of heterochromatin transcript levels at *cen-dg* (pericentromeric repeats), *mat3M::ura4^+^* (mating type locus) and *tlh1*^+^ (subtelomeric gene) by RT-qPCR. Transcript levels, normalized against *act1*, are presented relative to the WT median value (*n* = 8 independent biological replicates). (**E**) ChIP-qPCR analysis of H3K9me2 levels at constitutive heterochromatin domains (*cen-dg*, *mat3M::ura4*^+^ and *tlh1*^+^) and at facultative heterochromatin islands (*ssm4^+^*, *mei4^+^* and *SPBC24C6.09c*). Input-normalized IP samples are normalized to the average of two euchromatic loci (*act1^+^* and *tef3^+^*) and shown relative to the WT median value (*n* = 7 and three independent biological replicates, respectively). (**F**) Schematic representation of heterochromatin pathways involving the RNAi machinery, SHREC and Cul4-Ddb1^Cdt2^. Created in BioRender. Braun, S. (2024) *BioRender.com/z57f883*. (**G**) Quantification of heterochromatic transcripts from *cen-dg* repeats and the *mat3::ura4^+^* reporter gene by RT-qPCR. Transcript levels, normalized against *act1*, are presented relative to the WT median values (*n* = 3–4 independent biological replicates). (**H**) ChIP-qPCR analysis of H3K9me2 enrichment at *cen-dg* repeats and the *mat3::ura4*^+^ reporter gene. Input-normalized IP samples were normalized to the average of two euchromatic loci (*act1^+^* and *tef3^+^*) and are shown relative to the WT median value (*n* = 3–4 independent biological replicates). For (D), (E), (G) and (H), the individual replicates are displayed in box whisker or floating bar plots; the line depicts the median. For (G) and (H), statistical analysis was performed using one-way ANOVA tests, with letters denoting groups with significant differences as determined by Tukey’s post-hoc tests at *P* < 0.05.

Among the mutants displaying broad heterochromatin defects in Cluster II (Figure 1C), we uncovered *dhm2* (deleterious haploid meiosis), encoding an uncharacterized protein of 11.25 kDa. Dhm2 is highly conserved among the *Schizosaccharomyces* clade (Figure 4A). Secondary structure prediction data from the AlphaFold consortium (84) suggest that it consists of two alpha helices (Figure 4B). Dhm2 was previously identified in a sensitized genetic screening approach for mutants defective in heterochromatin maintenance at the mating type locus (67). However, its role in heterochromatin silencing remains unknown.

We therefore investigated the impact of Dhm2 on gene expression and the structural integrity of diverse heterochromatin domains. Using individual reporter growth assays and RT-qPCR analysis at the mating type locus, where the phenotype was most pronounced in data from our large-scale reporter assays, we confirmed the silencing defect in *dhm2*Δ cells, observing a 10-fold increase in *ura4 +* gene expression (Figure 4C and D). Endogenous heterochromatic transcripts from pericentromeric repeats and subtelomeres were moderately increased (Figure 4D), corroborating the broad role of Dhm2 in heterochromatin silencing. To probe whether loss of Dhm2 affects heterochromatin structure, we performed chromatin immunoprecipitation with H3K9me2- and H3K9me3-specific antibodies. ChIP-qPCR revealed a partial H3K9me2 decrease at various constitutive heterochromatin domains, while H3K9me3 remained unaltered (Figure 4E and Supplementary Figure S10a). The absence of Dhm2 had a stronger effect at facultative heterochromatin, leading to a nearly complete loss of H3K9me2 at *ssm4^+^* and *mei4^+^*, along with several other heterochromatin islands whose assembly requires the RNA processing and elimination factor MTREC (Figure 4E and Supplementary Figure S10b). In contrast, at other heterochromatin islands that are MTREC-independent but require Taz1 for assembly (e.g. *SPBC24c6.09*), H3K9me2 levels were unaffected or even increased (Figure 4E). Together, this implies a critical role of Dhm2 at specific facultative heterochromatin regions, whereas it appears to act redundantly with other pathways at constitutive heterochromatin domains.

To explore further a potential redundant role of Dhm2 in heterochromatin silencing, we introduced *dhm2*Δ into mutants lacking factors of known heterochromatin pathways (Figure 4F). When combining *dhm2*Δ with deficiency in RNAi (*ers1*Δ, *tas3*Δ), histone deacetylation (*clr3*Δ) or Epe1 degradation (*cdt2*Δ), we observed a strong synthetic defect in silencing (Figure 4G and Supplementary Figure S10c). In accordance with the aggravated silencing defects, H3K9me2 was virtually lost in these double mutants (Figure 4H and Supplementary Figure S10d). Together, these findings imply that Dhm2 contributes to heterochromatin silencing independently of these heterochromatin pathways.

### Dhm2 is required for heterochromatin maintenance

**Figure 5.**
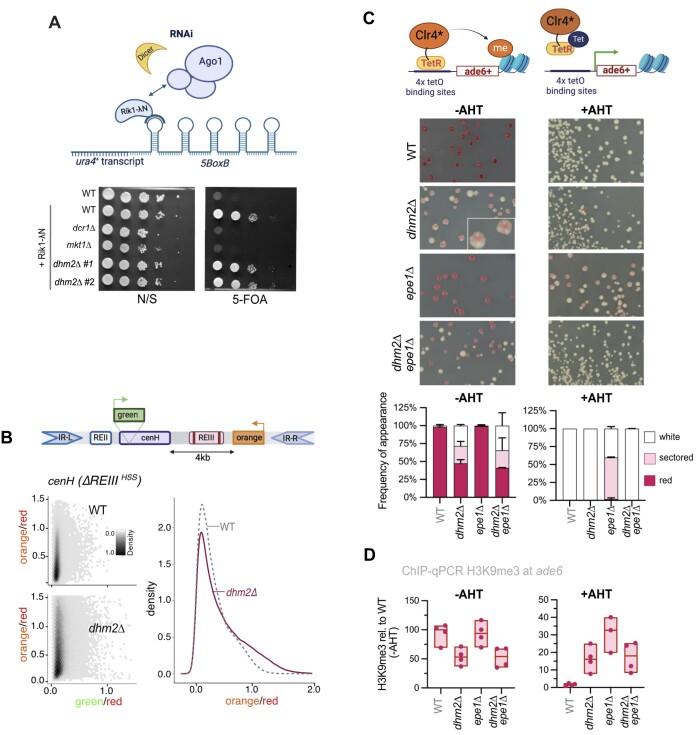
Dhm2 is required for heterochromatin maintenance. (**A**) Monitoring of RNAi-dependent heterochromatin establishment. Top: Schematic of the Rik1 tethering system to nascent transcripts via boxB binding sites at the 3′UTR of *ura4*^+^. Bottom: Silencing assay using the *ura4^+^-5BoxB* reporter. Serial 10-fold dilutions of strains expressing Rik1-ΔN, including wild-type (WT), positive controls (*dcr1*Δ, *mkt1*Δ) and two independent *dhm2*Δ strains, were plated on non-selective (N/S) and 5-FOA-containing media. The *rik1*^+^ strain expresses the non-fusion variant (negative control). (**B**) Monitoring of heterochromatin spreading at the silent mating type locus. Top: Schematic of the Δ*REIII^HSS^* (heterochromatin spreading sensor) system, with reporters inserted at *cenH* (‘green’; nucleation site), downstream of the REIII element (‘orange’; sensor site) and mutations in the Atf1/Pcr1 binding sites of REIII element (Δ*REIII^HSS^*) denoted by two vertical lines. An additional reporter gene (‘red’) is placed downstream of IR-R as a transcriptional noise filter (not shown). Bottom left: 2D hexbin plots display expression of ‘green’ and ‘orange’ reporters (normalized against ‘red’ expression), in WT and *dhm2*Δ mutant in the Δ*REIII^HSS^* the reporter strain. Right: Density plot showing red-normalized ‘orange’ reporter expression cells filtered for ‘green’ off state. (**C**) Monitoring of heterochromatin establishment and maintenance at an ectopic heterochromatin locus. Top: Schematic of the inducible TetR-Clr4* establishment system at the *ade6^+^* locus. Middle: Representative images of colony color assay. WT, *dhm2*Δ, *epe1*Δ and *dhm2*Δ *epe1*Δ carrying *4xtetO-ade6^+^* and expressing TetR-clr4* were grown on PMG with low adenine in the presence or absence of anhydrotetracycline (AHT). Bottom: Percentage of red-pink colonies, sectored colonies and white colonies. Mean values and the range (error bars) from two independent experiments are shown (numbers of cells examined from two experiments for -AHT: WT = 287/49, *dhm2*Δ = 463/480, *epe1*Δ = 213/108, *dhm2*Δ *epe1*Δ = 1175/605; for + ATH: WT = 511/72, *dhm2*Δ = 596/516, *epe1*Δ = 245/118, *dhm2*Δ *epe1*Δ = 458/553). (**D**) ChIP-qPCR analysis of H3K9me3 enrichment at ade6 locus. Input-normalized IP samples, normalized to the average of two euchromatic loci (*act1^+^* and *tef3^+^*), and are shown relative to the WT median value in the absence of AHT (*n* = 3–4 independent biological replicates). Schemes in (A), (B) and (C) created in BioRender. Braun, S. (2023) *BioRender.com/x35u872*; Braun, S. (2024) *BioRender.com/u10z983* and Braun, S. (2023) *BioRender.com/h31c876*, respectively.

Perturbation in gene silencing due to the absence of Dhm2 could result from defects in either heterochromatin establishment, spreading or maintenance. Initiation of heterochromatin establishment involves DNA-binding factors or RNAi (1). To determine whether Dhm2 is required for RNAi-mediated silencing, we employed the previously described Rik1-λN/boxB reporter. Heterochromatin assembly is triggered through the recruitment of the RITS complex by Rik1, which requires an intact RNAi-machinery (85). In this system, Rik1 is fused to the λN peptide recognizing boxB-binding elements sites integrated at the 3′-UTR of the *ura4* mRNA (ura4-5boxB, Figure 5A). As positive controls, we used mutants lacking Dcr1 or Mkt1, previously shown to be required for RNAi-dependent heterochromatin establishment (86). In contrast to *dcr1*Δ and *mkt1*Δ, the loss of Dhm2 did not disrupt *ura4^+^* silencing (Figure 5A). This result implies that Dhm2 is dispensable for RNAi-mediated post-transcriptional silencing, consistent with the above conclusion that Dhm2 acts redundantly with RNAi (Figure 4G and H).

To further investigate the role of Dhm2 in RNAi-dependent and -independent heterochromatin establishment and spreading, we conducted assays at various heterochromatin loci. The MAT locus is a well-established region where heterochromatin is nucleated by the *cenH* element (homologous to the RNAi-nucleated pericentromeric *cen-dh* fragment) and the RNAi-independent *REIII* element. Both elements independently recruit the H3K9 methyltransferase Clr4, and once nucleated, heterochromatin spreads convergently from each element and is redundantly maintained by different pathways (15,87). However, mono-nucleated spreading can be studied by inactivating the *REIII* nucleation site so that spreading is initiated solely by the *cenH* element (Figure 5B). To examine nucleation and spreading, we employed the previously established heterochromatin spreading sensor (HSS) system, which uses three distinct fluorescent reporter genes inserted at a nucleation site (‘green’), a sensor site (‘orange’) and an unrelated locus outside heterochromatin for normalization (‘red’; see above) (48). This system can be used to measure both heterochromatin establishment at the nucleation site (OFF or ON state of ‘green’) and heterochromatin spreading by determining the ratio between the repressed state of ‘green’ (OFF) and ‘orange’ (ON or OFF). We employed the HSS system to study heterochromatin at different domains, including the mating type locus (*ΔREIII^HSS^*), subtelomeres (*SUB^HSS^*) and an ectopic locus (*EC^HSS^*) (see ‘Materials and methods’ section).

We initially examined the ‘green’ reporter at the nucleation site in the ΔREIII^HSS^ strains to elucidate the role of Dhm2 in heterochromatin establishment. In *dhm2*Δ cells, we noted a minor population with increased green signal in comparison to the corresponding WT strain (Supplementary Figure S11a). This subtle effect was also evident in other domains, including two subtelomeric loci (∼11 and ∼37 kb upstream of the telomeric repeats) and an ectopic region, where heterochromatin is assembled via insertion of a pericentromeric *dh* element (Supplementary Figure S11a). To further investigate the influence of Dhm2 on heterochromatin spreading at the mating type locus, we analyzed the behavior of ‘orange’ in ΔREIII^HSS^ cells in which the green signal was OFF (indicating proper nucleation). Under this condition, a small subpopulation in ΔREIII^HSS^ cells lacking Dhm2 gained the orange signal (Figure 5B). Together, these findings may suggest a minor contribution of Dhm2 to heterochromatin establishment and spreading. However, given the subtle nature of these changes, this may not be the primary cause of the global silencing defects we observe in the absence of Dhm2.

Therefore, we examined whether Dhm2 functions in heterochromatin maintenance using an inducible heterochromatin establishment assay that allows monitoring of heterochromatin maintenance once the initial trigger mediating establishment has been switched off (49). In the absence of anhydrotetracycline (-AHT), the TetR-Clr4* fusion protein is recruited to *4xtetO* binding sequences, resulting in the silencing of the adjacent *ade6^+^* reporter gene and, consequently, cells turning red on media containing low adenine. Conversely, upon the addition of tetracycline (+AHT), TetR-Clr4* dissociates from the *4xtetO* site, allowing *ade6* expression and formation of white colonies due to H3K9me turnover, promoted by the putative demethylase Epe1 (49). We observed that, under establishment condition (-AHT), loss of Dhm2 resulted in increased white colony formation (>30%, Figure 5C). Many of the remaining red-pinkish *dhm2*Δ colonies exhibited a sectored phenotype (see enlargement, Figure 5C), akin to mutants with defective clonal propagation of heterochromatin during cell division (37,69). Notably, the appearance of white colonies and the sectored phenotype was also seen in the *dhm2*Δ *epe1*Δ double mutant. Under the maintenance condition (+AHT), both WT and *dhm2*Δ cells displayed 100% white colonies (Figure 5C), consistent with previous reports that heterochromatin at this locus cannot be maintained in the presence of Epe1 that counteracts H3K9me (49,88). While the repressed state was partially retained in *epe1*Δ cells (65% pinkish-sectored colonies), this was not observed in the *dhm2*Δ *epe1*Δ double mutant (100% white colonies), suggesting that Dhm2-dependent heterochromatin maintenance is independent of Epe1.

To investigate the chromatin structure at this inducible heterochromatic locus, we conducted H3K9me2 and H3K9me3 ChIP experiments. As anticipated by the previous study (49), we observed robust enrichment for both repressive marks in WT cells under establishment conditions (-AHT), resembling endogenous heterochromatin. This enrichment extended over 10 kb from the nucleation site and diminished to base-line levels when cells were cultured in the presence of AHT (Supplementary Figure S11b,c). We next analyzed H3K9me3, primarily associated with epigenetic inheritance (89), at the *ade6* locus in *dhm2*Δ, *epe1*Δ and the double mutant. In line with the appearance of white and sectored colonies in the growth assay, we detected a significant reduction in H3K9me3 levels (∼50%) in the *dhm2*Δ and *dhm2*Δ *epe1*Δ mutants under establishment conditions (Figure 5D). Notably, while residual H3K9me3 levels (15–20%) were still present in *dhm2*Δ and *dhm2*Δ *epe1*Δ cells grown with AHT, these levels were lower than in the *epe1*Δ mutant (30–35%). Taken together, these findings indicate that Dhm2 plays a critical role in heterochromatin establishment and maintenance at this ectopic locus.

### Loss of Dhm2 results in the induction of replication stress markers

Previous studies have identified various DNA replication factors contributing to heterochromatin maintenance (66,68,90) and linked them to the transfer of parental histones across the replication fork, ensuring epigenetic transmission of the repressive state (91–94). Our study also identified several replication mutants (*mcl1, pof3, pob3, mrc1*) that displayed phenotypic profiles similar to *dhm2*Δ (Figure 1C). We further noticed that the *dhm2*Δ exhibits more elongated cells (Supplementary Figure S12a), a cell phenotype often observed in mutants with replication defects (95). To explore the role of Dhm2 during replication, we tested if the *dhm2*Δ mutant shows defects in resuming DNA replication upon transient replication stress. Cells were arrested in early S-phase by 4 h of HU (hydroxy urea) treatment at 20 mM and then released into HU-free media. While DNA content analysis by FC revealed that WT and *dhm2*-deleted strains progressed through S-phase similarly, chromosome analysis by PFGE showed that *dhm2*Δ has a slight defect in completing DNA replication upon release from HU (Supplementary Figure S12b). Compared to the asynchronous condition, the intensity of migrating chromosomes is twice as high 90 min after release in WT but not in *dhm2*Δ cells. This indicates that Dhm2 is required to promote chromosome duplication after transient fork stalling suggesting that Dhm2 plays a role in promoting DNA replication upon replication stress.

**Figure 6.**
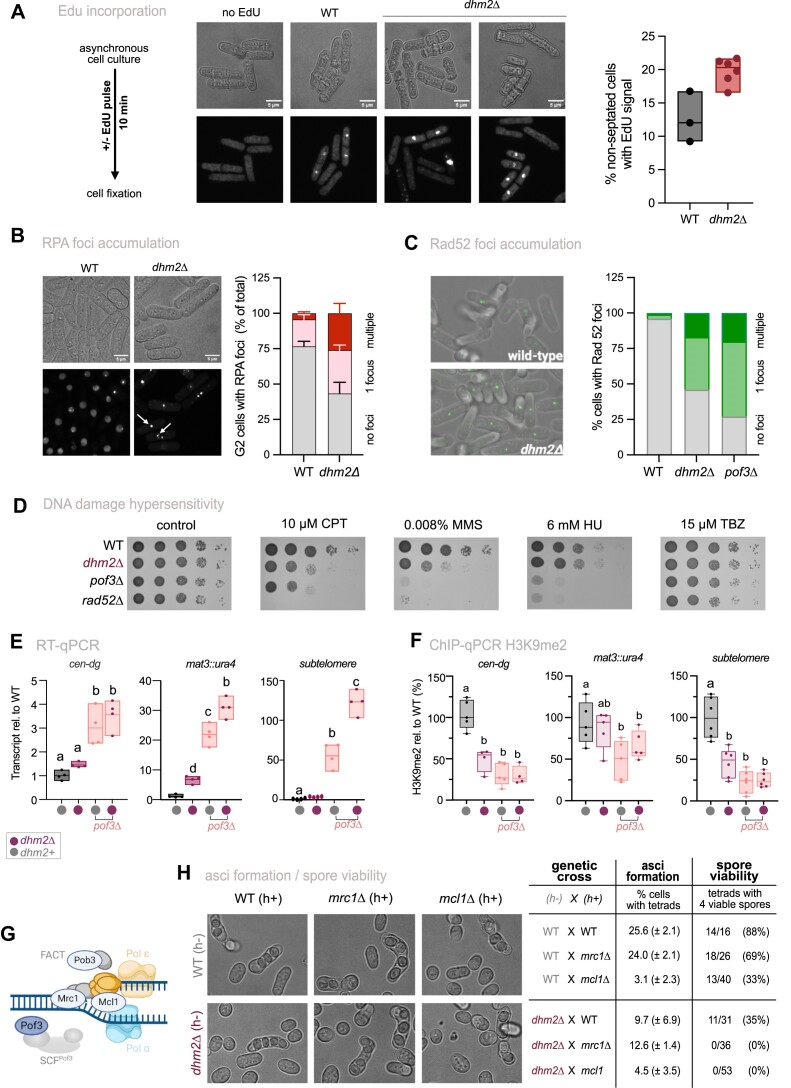
Loss of Dhm2 results in induction of replication stress markers. (**A**) Quantification of DNA synthesis outside S-phase. DNA synthesis in non-septated cells was detected by fluorescence microscopy following a short pulse of EdU. (**B**) Accumulation of RPA foci. Left panel: Representative images Ssb3-YFP foci in WT and *dhm2*Δ cells. Right panel: Percentage of cells with RPA foci formation. Shown are mean values and errors (95% confidence interval) from 3 (WT) and 7 (*dhm2*Δ) biological replicates. (**C**) Accumulation of Rad52-GFP foci. Left panel: Representative images of WT and *dhm2*Δ cells expressing Rad52-GFP. Right panel: Percentage of Rad52-GFP foci formation in WT, *dhm2*Δ and *pof3*Δ cells. (**D**) Sensitivity to DNA damage. Ten-fold serial dilutions of the indicated strains were plated on YES medium supplemented with different DNA-damaging agents (HU, hydroxyurea; CPT, camptothecin; MMS, methyl methanesulfonate) and incubated for 3 days at 32°C. (**E**) RT-qPCR analysis of heterochromatic transcripts (*cen-dg, mat3M::ura4*^+^ and *tlh1*^+^) in the indicated strains. Transcript levels, normalized against *act1*, are presented relative to the WT median value (*n* = 4 independent biological replicates). (**F**) ChIP-qPCR analysis of H3K9me2 levels at heterochromatin domains (*cen-dg*, *mat3::ura4*^+^ and *tlh1^+^*) in the indicated strains. Input-normalized IP samples, normalized to the average of two euchromatic loci (*act1^+^* and *tef3^+^*), are shown relative to the WT median value (*n* = 4–6 independent biological replicates). (**G**) Scheme illustrating factors involved in DNA replication and epigenetic inheritance. Created in BioRender. Braun, S. (2023) *BioRender.com/z39c488*. (**H**) Ascus formation efficiency and spore viability of dhm2 single and double mutants. Representative images of asci formed 3 to 4 days after mating (*h^+^* strains, top: WT, *mrc1*Δ, *mcl1*Δ; *h^−^* strains, left: WT, *dhm2*Δ). Table shows the genetic cross scheme (left), percentage of cells with complete tetrads (middle) and the number of tetrads with four viable spores after germination on YES media (right). For quantification of ascus formation, mean value and deviation (range) are presented from two independent experiments (∼300 cells analyzed per cross).

Upon replication stress, DNA synthesis can occur outside the bulk of S-phase (septated cells) to complete DNA replication before cells enter mitosis (96). We therefore tested whether cells lacking Dhm2 suffer from endogenous replication stress manifested as more cells undergoing DNA synthesis outside the S-phase. DNA synthesis was detected by incorporation of a thymidine analog that contains an alkyne group, which can be conjugated to a fluorescently labeled azide in a Cu(I)-catalyzed ‘click’ reaction. Cells from asynchronous populations were exposed to a short pulse of EdU (150 μM), and DNA synthesis upon EdU incorporation was detected by fluorescence microscopy (Figure 6A). Non-S-phase cells were distinguished by the absence of septum. Compared to WT, deletion of *dhm2* led to an increase of the number of non-S-phase cells undergoing DNA synthesis. This indicates that DNA synthesis occurs more frequently outside of the bulk of S-phase in the absence of Dhm2, as a consequence of incomplete DNA replication at the end of S-phase. The analysis of RPA foci, a marker of single-stranded DNA (ssDNA), further confirmed signs of endogenous replication stress in the absence of Dhm2: First, deleting *dhm2* increased the percentage of G2 cells with Ssb3-YFP foci, a subunit of Replication Protein A (RPA) complex (Figure 6B). Second, whereas most WT cells exhibited a single and discrete RPA focus, *dhm2*Δ cells often showed multiple RPA foci or a mega RPA focus, a replication stress feature previously observed in replication mutants (97).

The RPA-coated ssDNA is a key signaling platform that activates the checkpoint kinase ATR/Rad3 to protect and repair replication forks during replication stress (97). We therefore tested if repair factories are also increased in the *dhm2* mutant. Rad52 (also known as Rad22 in *S. pombe*) plays a key role in DNA repair by homologous recombination (98). Upon induction of DNA double-strand breaks, Rad52 binds to single-stranded DNA, forming distinct nuclear foci (99). Live-cell imaging revealed that *dhm2*Δ displayed elevated levels of Rad52 foci in >50% of cells (Figure 6C), indicating that double-strand DNA breaks accumulate in the absence of Dhm2. This phenotype was similar to a mutant lacking the F-box protein Pof3^Dia2^ involved in the degradation of DNA polymerases and other replication factors (100–104). Interestingly, Rad52 was also identified by our screens, and *rad52*Δ cells had a silencing defect at *MAT* (Cluster VI, Supplementary Figure S6a and Supplementary Figure S12c). Many replication mutants are sensitive toward genotoxic agents affecting various steps during replication fork progression. We found that *dhm2*Δ is sensitive toward the topoisomerase inhibitor camptothecin (CPT) and the DNA alkylation agent methyl-methane sulfonate (MMS), partially phenocopying the sensitivity of the *pof3*Δ mutant (Figure 6D). In contrast, *dhm2*Δ was insensitive toward HU and thiabendazole (TBZ), an inhibitor of microtubule formation affecting mitotic progression.

Genes involved in shared pathways often exhibit epistatic genetic interactions. Intriguingly, while pericentromeric transcripts were more abundant in the pof3Δ mutant than in the *dhm2*Δ mutant, the *dhm2*Δ *pof3*Δ double mutant displayed a non-additive defect in transcript levels (Figure 6E). This epistatic interaction was not observed at other heterochromatic domains (mating type locus, subtelomeres) in the double mutant, which exhibited an additive or even synergistic increase in heterochromatic transcript accumulation. However, the reduction in H3K9me2 levels in the double mutants was non-additive and comparable to that of the corresponding single mutants for all heterochromatin domains examined (Figure 6F). This contrasts the synthetic defects observed in *dhm2*Δ when combined with other mutants (e.g. RNAi and SHREC) (Figure 4H).

Previous studies have implicated other factors associated with the replication fork, such as Mcl1 and Mrc1 (Figure 6G) in heterochromatin silencing, which our findings corroborate (Figure 1C and Supplementary Figure S12d). However, efforts to generate viable *dhm2*Δ *mrc1*Δ or *dhm2*Δ *mcl1*Δ double mutants through genetic crosses using random spore analysis or tetrad dissection were unsuccessful, suggesting a synthetic lethal interaction. We noticed that *dhm2*Δ *and mcl1*Δ single mutants, when crossed with WT cells, displayed a reduced number of asci that contain a complete set of spores (10% and 3%, respectively, compared to 26% for crosses using WT cells only; Figure 6H). Interestingly, this reduction was not further exacerbated in the corresponding *dhm2*Δ *mrc1*Δ or *dhm2*Δ *mcl1*Δ double mutants, indicating that tetrad formation was still possible. However, spore germination was dramatically impaired in the double mutants, leading overall to a decrease in viable spores compared to the single-mutant crosses (Figure 6H). These results further reinforce the functional link between Dhm2 and DNA replication.

Collectively, our findings demonstrating that Dhm2 loss causes endogenous replication stress and hypersensitivity towards DNA damage strongly support the notion that defects in heterochromatin silencing and inheritance in dhm2Δ cells may result from defective DNA replication and/or DNA repair.

## Discussion

In this study, we present a systematic and quantitative growth-based reporter approach that unveils numerous factors influencing silencing at the major constitutive heterochromatin domains in *S. pombe*. The comprehensive nature of our investigation not only revealed many new factors but also corroborated the roles of genes previously associated with heterochromatin silencing, aligning our results with independent studies. Beyond the identification of factors, our quantitative approach and different reporter systems allowed us to allocate these factors to distinct functional pathways that show specificity for different heterochromatin domains. Among the new factors, we unveiled a potential role in heterochromatin maintenance and DNA replication or repair for Dhm2, providing a functional link between DNA replication and heterochromatin inheritance. In the following, we explore the implications of these findings.

### A comprehensive collection of factors implicated in heterochromatin silencing

From a pool of 100 genome-wide datasets, we identified 180 mutants that displayed a significant decrease in heterochromatin silencing, employing stringent selection criteria based on threshold and reproducibility. Many of the candidates we identified were further validated by directly examining heterochromatic transcripts, revealing a substantial consistency between growth-based reporter assays and transcript levels (Supplementary Figure S3 and Supplementary Table S7). This collection of mutants represent a considerable increase in the number of candidates compared to previous studies employing the genome-wide gene deletion library from Bioneer. A key distinction in our approach was the use of multiple reporter strains to monitor silencing across different heterochromatin domains (*CEN*, *MAT*, *SUBTEL* and *TEL*). In contrast, prior studies conducted single reporter screens (63–68). Other critical factors contributing to the large number of candidates identified by our study include the use of quantitative measurements involving both technical replicates and multiple independent screening rounds, normalization of reporter assays, and the integration of two distinct readout methods for monitoring reporter activity (+FOA and −URA). This advanced approach provided our study with high robustness, reproducibility, and increased sensitivity in the context of reporter readouts.

In a few cases, we noticed some deviations between growth-based reporter assays and endogenous transcript levels. These may arise from differences in the experimental setup or sensitivity of assays, or could also be attributed to cellular heterogeneity. Using assays that enable single-cell detection, we could indeed segregate cellular subpopulations with different heterochromatin states (Figure 3).

Upon comparing published datasets with our study, we observed striking similarities with respect to specificity and overlapping functions of factors across heterochromatin domains. When consolidating all genes accumulated from previous studies, we found that approximately half of them (57 out of 120 genes) were also identified through our screens. It is noteworthy that the heterochromatin loci investigated in our study and previous reports were not entirely identical (Supplementary Figure S5). While we investigated the pericentromeric *imr1L* repeats and a subtelomeric locus 7 kb upstream of telomeres, the other studies focused on the *cen-dg* repeats (63,64) and the *SPAC212.07* gene 24 kb upstream of telomere (68), respectively. This experimental divergence could explain the differences in identified candidates. In contrast, the majority of genes identified for mating type silencing at *mat2P-ΔREII* and *mat3M-EcoRV* loci (66,67) and telomeric repeats on the *Ch16* minichromosome (65) were also discovered by our study, consistent with the identical or similar arrangement of the reporter genes (i.e. *mat3M-EcoRV*, telomeric repeats on *TEL2L*). Thus, independent datasets and variations in reporter systems increase the overall fidelity of our understanding of heterochromatin silencing.

### Specificity and requirement of regulators at different heterochromatin domains

A striking observation was that most candidates affected specific subsets of heterochromatin domains, while other regions remained unaffected (Figure 1 and Supplementary Figure S5). This may reflect mechanistic differences in the establishment and maintenance of heterochromatin at those chromosomal regions. A notable example is RNAi, which is essential at pericentromeres but acts redundantly with additional pathways at other heterochromatin regions (15,17,38,105). Consistently, genes implicated in RNAi were exclusively identified through *CEN* reporter screens (Cluster VII; Figure 1 and Supplementary Figure S6). An exception is the *arb2* mutant, lacking a component of ARC (Argonaute siRNA chaperone) involved in siRNA maturation (106). While this mutant exhibits a uniform silencing defect across all heterochromatin domains, this might be caused by additional misregulation of the juxtaposed *crb3* gene, which overlaps with the 3′-UTR of *arb2^+^*. Intriguingly, Crb3 is a member of the RNA-processing Rix1 complex (RIXC), and the combined loss of RIXC and RITS causes synthetic silencing defects (37,40). Another example of redundancy can be observed with the novel silencing factor Dhm2, whose role in silencing at constitutive heterochromatin is partially obscured by members of the heterochromatin core machinery (e.g. RNAi and SHREC). In contrast, the absence of Dhm2 results in the complete loss of heterochromatin at sites of facultative or ectopically induced heterochromatin, implying the lack of compensatory mechanisms in these regions (as discussed below).

Heterochromatin domains may also vary in their specific needs in heterochromatin assembly. Spreading and maintenance of heterochromatin require mechanisms contributing to high nucleosome abundance and stability (48,69,107). Several factors promoting nucleosome stability (Fft3, Spt6, Abo1 and others) were enriched when selectively targeting silencing at the mating type locus (Cluster III and VI; Figure 1 and Supplementary Figure S6). Notably, these factors were also identified by previous studies employing similar mating-type specific approaches (66,67,69). While some of these factors have been reported to affect additional heterochromatin regions (66,108,109), the pronounced sensitivity of the mating type locus suggests that this chromosomal region heavily relies on factors ensuring stable heterochromatin maintenance. A distinguished feature of this heterochromatin domain is the presence of multiple, well-defined nucleation sites, such as the *REII* and *REIII* elements, from which heterochromatin spreads into neighboring regions. In contrast, silencing at heterochromatin regions that primarily rely on RNAi, like the pericentromeric *dg* repeats, appears to undergo continuous heterochromatin establishment driven by siRNAs (48). Hence, these distinct mechanisms may also account for the requirement of different factors for heterochromatin maintenance.

Another chromatin context-specific observation was the identification of numerous factors linked to metabolic pathways, including methionine and membrane lipid biosynthesis, specifically affecting silencing at subtelomeres and the mating type locus (Figure 3 and Supplementary Figure S9). While most of these factors were missed by the previous genome-wide studies, other research reported several links to methionine and SAM synthesis. Methylenetetrahydrofolate reductase Met11 plays a role in methionine regeneration through 5-MTHF generation and was noted to affect heterochromatin integrity (110). Additionally, SAM synthetase was among the SU(VAR) mutants displaying altered position effect of variegation in flies (111) and was further shown to be crucial for silencing and perinuclear heterochromatin anchoring in worms (112). Of note, in *S. pombe*, SAM synthetase is encoded by a single essential gene (113), explaining its absence in our study. Given SAM’s role in various methylation reactions, including H3K9me, methionine availability likely affects silencing in *S. pombe*, as observed in mammals (41). SAM is also essential for the biosynthesis of membrane phospholipids. Supporting the idea of nuclear membrane composition impacting silencing, our study identified two methyltransferases (Cho2, Cho1^Opi3^) involved in phosphatidylcholine synthesis. Previously, Cho2 was also shown to physically interact with Lem2, another inner nuclear membrane protein contributing to heterochromatin silencing through multiple functions (38,114–116). Thus, SAM deficiency appears to affect multiple pathways critical for silencing of subtelomeres (and to a lesser extent the mating type locus). Surprisingly, deficiencies in these pathways did not seem to impact pericentromeric heterochromatin (Figure 3). As different heterochromatin domains compete for shared pools of silencing factors (38,76), this may suggest that subtelomeres are less favorable sites for heterochromatin assembly compared to other regions. Alternatively, the absence of defined boundary elements and the gradual decline of H3K9me-marked heterochromatin toward telomere-distal regions may cause subtelomeres to acquire dispersed heterochromatin structures, making them more vulnerable to fluctuations in the supply of factors required for their assembly, like SAM. In agreement with the chromatin source-sink hypothesis (117), both scenarios are supported by the notion that subtelomeres can function as sinks for extra silencing factors, whereas the buffering capacity at pericentromeres is restricted by the presence of strict boundaries (76,118).

### Unveiling additional functions of heterochromatin regulators

Our systematic approach comprehensively captured previously known architectural and functional submodules in protein complexes involved in chromatin organization (Figure 2). However, we also identified notable exceptions, suggesting additional roles for these factors. This was evident for complex members of SAGA (Sgf29, Sgf73), DASH (Spc19) or Mms19, a component of the cytosolic iron-sulfur assembly (CIA) machinery (Figure 2 and Supplementary Figure S7). The DUB member Sgf73 was indeed shown to promote RITS assembly independently of its role within SAGA (81). Proteins from other complexes may share similar ‘moonlighting functions’ or play multiple roles in heterochromatin silencing. For instance, Mms19 has been linked to DNA metabolism and methionine biosynthesis (119–121), but also associates with Cdc20 and Rik1-Raf2, factors linked to DNA replication and H3K9 methylation (90). While these functions may not be mutually exclusive, the phenotypic profile of *mms19 Δ* resembles more closely those of mutants deficient in methionine biosynthesis (Figure 1, Cluster I versus V). Moreover, Cdc20 possesses a 4Fe-4S cluster in its catalytic domain, suggesting that it might be a substrate of Mms19 which inserts Fe-S into apo-proteins (122). Therefore, further work is necessary to discern whether Mms19 functions directly (Rik1-Raf2 interaction) or indirectly (Fe-S cluster assembly) in heterochromatin maintenance.

Consistent with a previous proteomics study (74), our study identified two nucleoporins (Npp106 and Nup132) and we confirmed their role in silencing heterochromatic transcripts (Supplementary Figure S6). While a broader involvement of nucleoporins in heterochromatin organization has been proposed, other inner nuclear membrane proteins present in Swi6^HP1^-purified heterochromatin did not appear to be crucial for silencing (Supplementary Table S3 and Supplementary Figure S6) (74). Recently, Nup132 has been implicated in recruiting the SUMO protease Ulp1 to de-SUMOylate Lem2, a regulatory switch crucial for its role in silencing (38,123). Cells lacking Nup132 or Lem2 display silencing defects under rich growth conditions but not in minimal media, as used in our study (115,123,124), explaining the absence of Lem2 in our current candidate list. The mechanism allowing cells to bypass Lem2 under certain conditions remains unclear, but this finding underscores the adaptability and dynamic regulation of heterochromatin pathways in diverse environmental conditions. Nevertheless, the retrieval of Nup132 in our study suggests additional functions in silencing. Thus, further exploration is needed to gain a comprehensive understanding of the role of nucleoporins in heterochromatin silencing.

### Role of Dhm2 and replication factors in heterochromatin inheritance

Among mutants affecting all heterochromatin domains, we discovered Dhm2, a protein of unknown function previously identified in a genetic screen for mating-type locus silencing defects (67). Given the lack of known protein motifs, the mechanism through which Dhm2 contributes to heterochromatin silencing remains unclear. We demonstrate that Dhm2 acts redundantly with various common silencing pathways at constitutive heterochromatin (Figure 4), providing a rationale for the moderate silencing defects observed in the single mutant. This may also explain why *dhm2*Δ had not been retrieved by most other studies.

Dhm2 may be involved in specific steps during heterochromatin assembly (i.e. nucleation, spreading or maintenance). We found that Dhm2 is largely dispensable for RNAi- and shelterin-dependent heterochromatin establishment, consistent with acting redundantly rather than through these pathways. While we did not explore other possible RNA- or DNA-dependent establishment mechanisms, the broad involvement of Dhm2 at diverse heterochromatin domains makes a specific role in establishment less likely. We also observed only a modest effect on heterochromatin spreading when examining the mating-type locus (125). However, Dhm2 had a substantial impact on heterochromatin maintenance at an ectopic locus where heterochromatin assembly can be induced independently of RNAi (Figure 5). Although silencing at this locus was compromised under both heterochromatin establishment and maintenance conditions, two critical observations suggest Dhm2 primarily contributes to the latter. First, under heterochromatin establishment conditions, red colonies (repressed reporter gene) often displayed a red-white sectoring phenotype, indicating that heterochromatin cannot be stably maintained without Dhm2. This variegating phenotype, shared by many mutants deficient in heterochromatin maintenance (37,40,69), suggests that heterochromatin is not properly inherited during cell division. The appearance of white colonies may further imply a quick turnover of heterochromatin rather than a defect in the initial establishment. Second, although the absence of Epe1 allows silencing even under maintenance conditions (i.e. when *de novo* heterochromatin assembly is absent), silencing in *epe1*Δ cells appeared to be completely lost by the additional lack of Dhm2, resulting in the exclusive appearance of white colonies. While *dhm2Δ epe1*Δ cells retained residual amounts of H3K9me3 under this condition, the levels were lower than in epe1Δ cells. These findings not only imply that Dhm2 is critical for heterochromatin maintenance but also that it acts independently of Epe1. Remarkably, unlike WT cells, the *dhm2*Δ single mutant also maintained low H3K9me3 levels, a phenomenon that coincided with the extremely rare appearance of red colonies. These intriguing finding warrants further investigation in future studies.

The loss of heterochromatin maintenance in *dhm2*Δ may result from defects in epigenetic inheritance during DNA replication. Several lines of evidence support a role for Dhm2 in replication. First, *dhm2*Δ cells exhibit elongated morphology and struggle to complete DNA replication at the end of S-phase. Second, they accumulate single-stranded DNA and Rad52 foci indicating the formation of DNA breaks. Third, *dhm2*Δ cells are hypersensitive to various DNA-damaging agents (Figure 6). Notably, in line with prior reports (66,68,90,94), we identified various replication factors, although they differed in the extent of silencing defects and specificity of the affected heterochromatin domains (Supplementary Figure S12). The similarities in phenotypic profiles (Figure 1) and the partially non-additive genetic interactions of Dhm2 and the F-box protein Pof3^Dia2^ (Figure 6) suggest that they act in similar pathways. As part of a ubiquitin ligase, Pof3^Dia2^ mediates the turnover of DNA polymerases and other replication factors (100–104). Furthermore, its absence causes hypersensitivity toward various DNA-damaging agents and accumulation of Rad52 foci indicating double-stranded DNA breaks, a phenotype partially shared by *dhm2*Δ (Figure 6). Thus, it is plausible that Dhm2 is directly involved in Pof3-related steps, although both may also have independent functions in different chromatin contexts, as indicated by the synergistic defects at subtelomeric heterochromatin. Dhm2 might contribute to other aspects of replication, as suggested by the synthetic lethality with cells lacking Mrc1 and Mcl1, components of the replication fork. Mrc1 has been shown to facilitate epigenetic inheritance by directly binding to the H3-H4 tetramer, promoting the transfer of parental histones. Notably, this histone-chaperone function is independent of its role in DNA replication (91–93). Additional work is needed to elucidate the specific function of Dhm2 during replication and determine whether its role in heterochromatin maintenance is separable from DNA synthesis.

Dhm2 is highly conserved within the *Schizosaccharomyces* species, but no homologs have been found outside this clade. However, its critical function in maintaining heterochromatin structures in the absence of redundant pathways makes it likely that similar mechanisms exist in other eukaryotes. The predicted α-helical structure and the lack of known protein motifs suggest that it acts as an adaptor, facilitating the interactions of partner proteins. Given its small size, it may be possible that structures similar to Dhm2 are integral components of other polypeptides in higher eukaryotes. Future work focused on identifying Dhm2 interaction partners and gaining insights into its proteome will elucidate its role in heterochromatin inheritance, its potential link to DNA replication, and the broader conservation of those mechanisms.

## Concluding remarks

Our present study provides critical insights into the diverse roles of functional pathways and their specific contributions to heterochromatin domains, a previously underexplored aspect of heterochromatin biology. By employing a combination of multiple, quantitative and sensitive reporter systems and phenotypic profiling, we have identified a plethora of heterochromatin regulators that often exhibit domain-specific silencing effects. This approach has also led to the discovery of numerous genes involved in metabolic pathways and elucidated the role of the newly identified regulator Dhm2 in heterochromatin maintenance. Furthermore, we have uncovered distinct functions within physical protein complexes, submodules or even individual subunits, suggesting the existence of ‘moonlighting’ activities for some of these factors. This wealth of knowledge represents a significant contribution to the field, offering valuable insights for future studies.

Despite the significant findings of our study, we recognize certain limitations. Genetic screens employing single mutants can only identify factors that play essential roles in silencing. The absence of a pronounced phenotype in a single mutant at a particular heterochromatin domain does not preclude its potential involvement in silencing. Thus, factors exhibiting domain-specific behaviors may also fulfill additional roles at other heterochromatin domains, masked by the presence of redundant pathways. Additionally, certain factors and pathways may be critical for silencing only under specific conditions. Finally, our study focused exclusively on constitutive heterochromatin domains, while other genomic regions, such as facultative heterochromatin, were excluded. This could explain the absence of conserved proteins implicated in regulating heterochromatin in other systems, including human cells (126). Therefore, we recommend future research incorporating follow-up screens utilizing additional reporters and combinatorial approaches, such as E-MAP (epistasis mini array profiling) and varying growth conditions, to address these limitations.

## Supplementary Material

gkae1024_Supplemental_Files

## Data Availability

The datasets supporting the conclusion of this article are included within the article and as Supplementary Tables S1-S14 as individual spreadsheets in a MS-Excel file. Additional data and R scripts are available on the zenodo repository: https://doi.org/10.5281/zenodo.11209900.

## Notes

Present address: Matias Capella, Instituto de Agrobiotecnología del Litoral, CONICET, Universidad Nacional del Litoral, Santa Fe, Argentina.

Present address: Ramón Ramos Barrales, Centro Andaluz de Biología del Desarrollo (CABD), Universidad Pablo de Olavide-Consejo Superior de Investigaciones Científicas-Junta de Andalucía, Seville, Spain.
