# Supplementary material for: A systematic quantitative approach comprehensively defines domain-specific functional pathways linked to Schizosaccharomyces pombe heterochromatin regulation
Source: Nucleic Acids Res. 2024 Nov 20;52(22):13665–89. doi: 10.1093/nar/gkae1024 (PMC11662645; doi:10.1093/nar/gkae1024)
Supplement: gkae1024_Supplemental_Files [file gkae1024_supplemental_files.zip › Suppl. Figures incl. legends.pdf]

Supplementary Figures

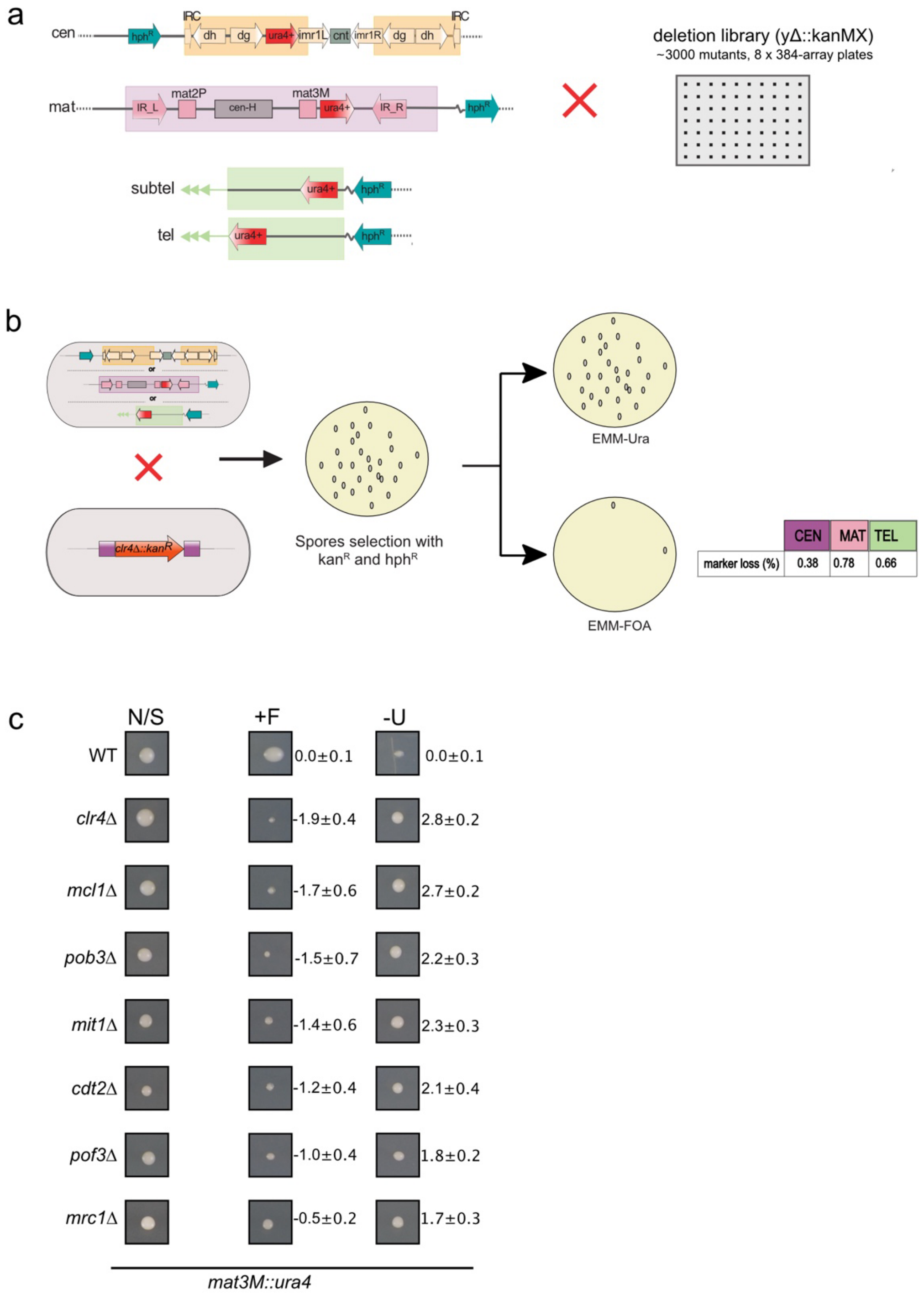

Figure S1. Genetic linkage of selection marker and reproducibility of silencing reporter assays. a) Schematic illustration of the *ura4*$^+$ reporter gene insertions at the four constitutive heterochromatic loci. Shaded areas represent heterochromatin areas, each differentiated by unique color codes. The selection marker (*hphMX6*), which confers hygromycin resistance, was placed 2-4 kb from the heterochromatin boundary within the euchromatin region, ensuring genetic linkage with the *ura4*$^+$ reporter gene. **b)** Genetic linkage analysis: Yeast strains carrying the *ura4*$^+$ reporter at various heterochromatic sites (*CEN, MAT, TEL*) were crossed to a *clr4*Δ strain lacking the H3K9 methyltransferase Clr4. Resulting spores that carry both *clr4*Δ and *hphR* marker were selected by replica-plating onto media containing 5-FOA (+F) or media lacking uracil (-U) minimal media to evaluate *ura4*$^+$ presence. Growth in the presence of 5-FOA indicates a loss of the genetic linkage between *ura4*$^+$ and *hphR.* Quantification of the genetic linkage tests is shown in the table. **c)** Reproducibility of silencing reporter assays. Displayed are representative colony images from a *MAT* locus screen. The media types are labeled as N/S (non-selective), +F (5-FOA-containing), and -U (uracil-deficient). Quantification of N/S-normalized colony sizes relative to wild-type are shown next to images as $\log_2$-transformed mean values with standard deviation (SD; (n = 7 biological replicates, each based on two technical replicates).

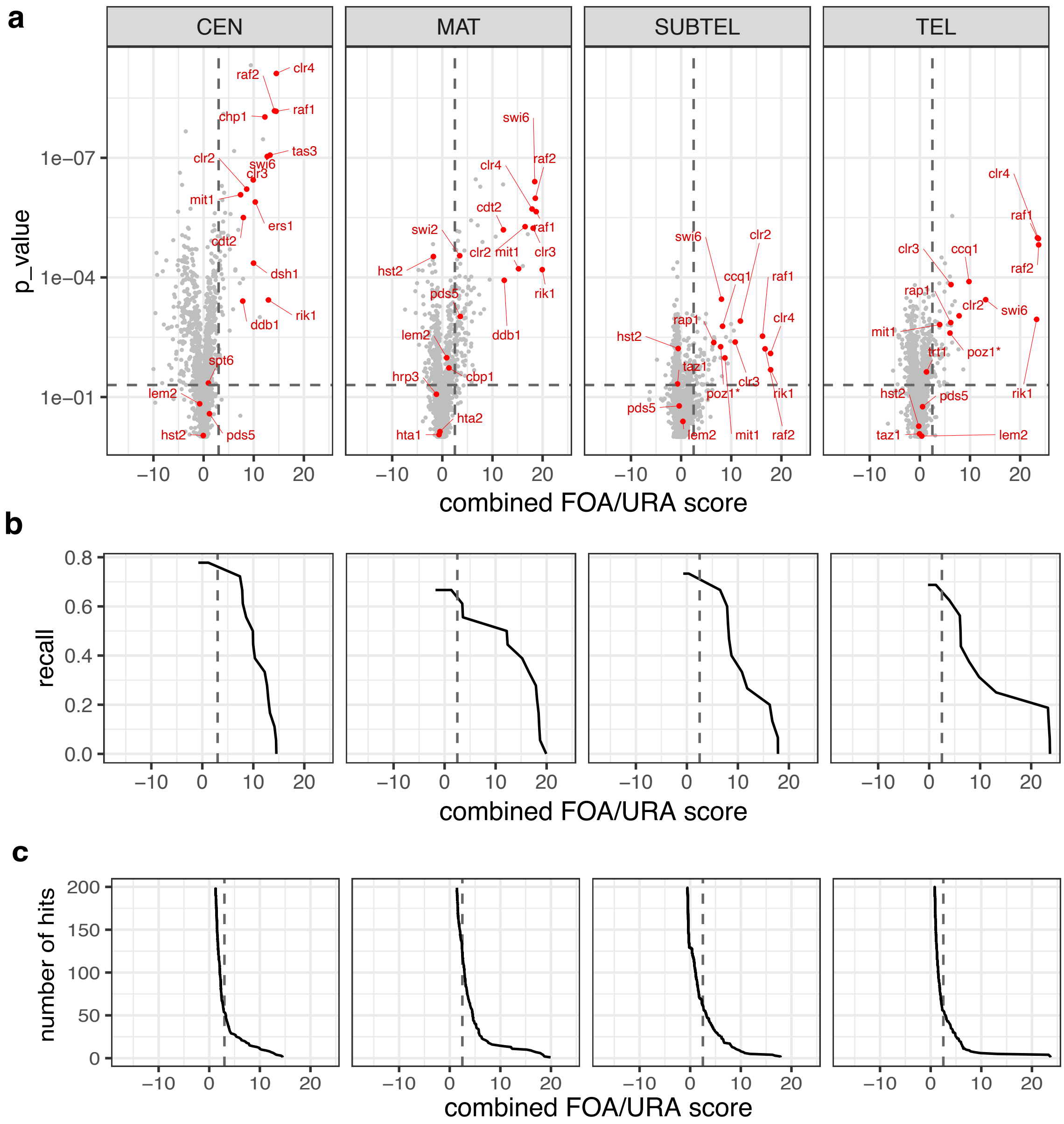

Figure S2. Retrieval of known heterochromatin factors dependent on threshold parameters (recall). a) Volcano plots displaying combined FOA/URA scores and reproducibility (P-value from one-sample Student's t-test) for all mutants from various screens. Prior FOA/URA score combination, relative growth scores for FOA and URA were z-normalized (i.e., setting standard deviation to 1) and median-centered for each heterochromatin reporter, ensuring comparability across all heterochromatic loci (see Methods). Red dots highlight known heterochromatin factors (refer to Table S4). The datasets comprise 3-8 independent biological replicates (*CEN*: 8; *MAT*: 7; *SUBTEL*: 3; *TEL*: 6; each with 2-4 technical replicates). **b)** Recall values of known heterochromatin factors. Plots showing screen sensitivity (ratio of the factors retrieved to the total number of factors) dependent on threshold setting for combined FOA/URA score. **c)** Hit counts relative to threshold settings. The plots show the number of hits dependent on the threshold settings. The dashed lines in (b) and (c) indicate the specific thresholds for the combined FOA/URA scores applied for hit identification: *CEN* = 3; *MAT, SUBTEL, TEL* = 2.5.

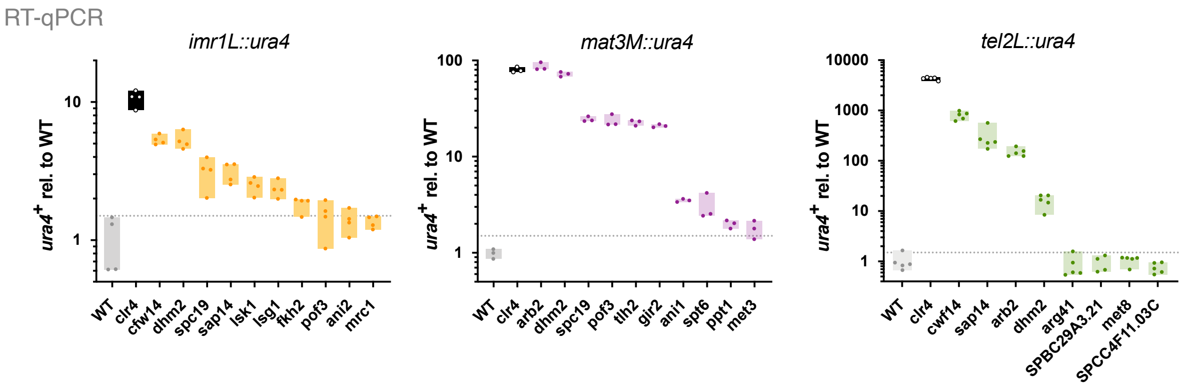

Figure S3. Screen validation by measuring *ura4*+ reporter transcript levels (precision). Transcript levels of the *ura4*$^{+}$ reporter gene, located at *imr1L, mat3M,* and *tel2L,* were quantified using RT-qPCR. Transcript levels were normalized against *act1* and are depicted relative to the WT mean values (n = 4 independent biological replicates). Presented is a representative subset of candidates identified based on the threshold settings employed in this study. The dashed line represents the threshold set for elevated expression (1.5-fold relative to WT). The results are summarized in Suppl. Table S6. Note that for a few candidates identified, *ura4*$^{+}$ transcripts were not found to be increased by RT-qPCR, resulting in precision scores < 1. These deviations might be attributed to differences in the experimental setup (solid minimal media vs. liquid rich media; clonal vs. population-based growth) or in the sensitivity of the assay.

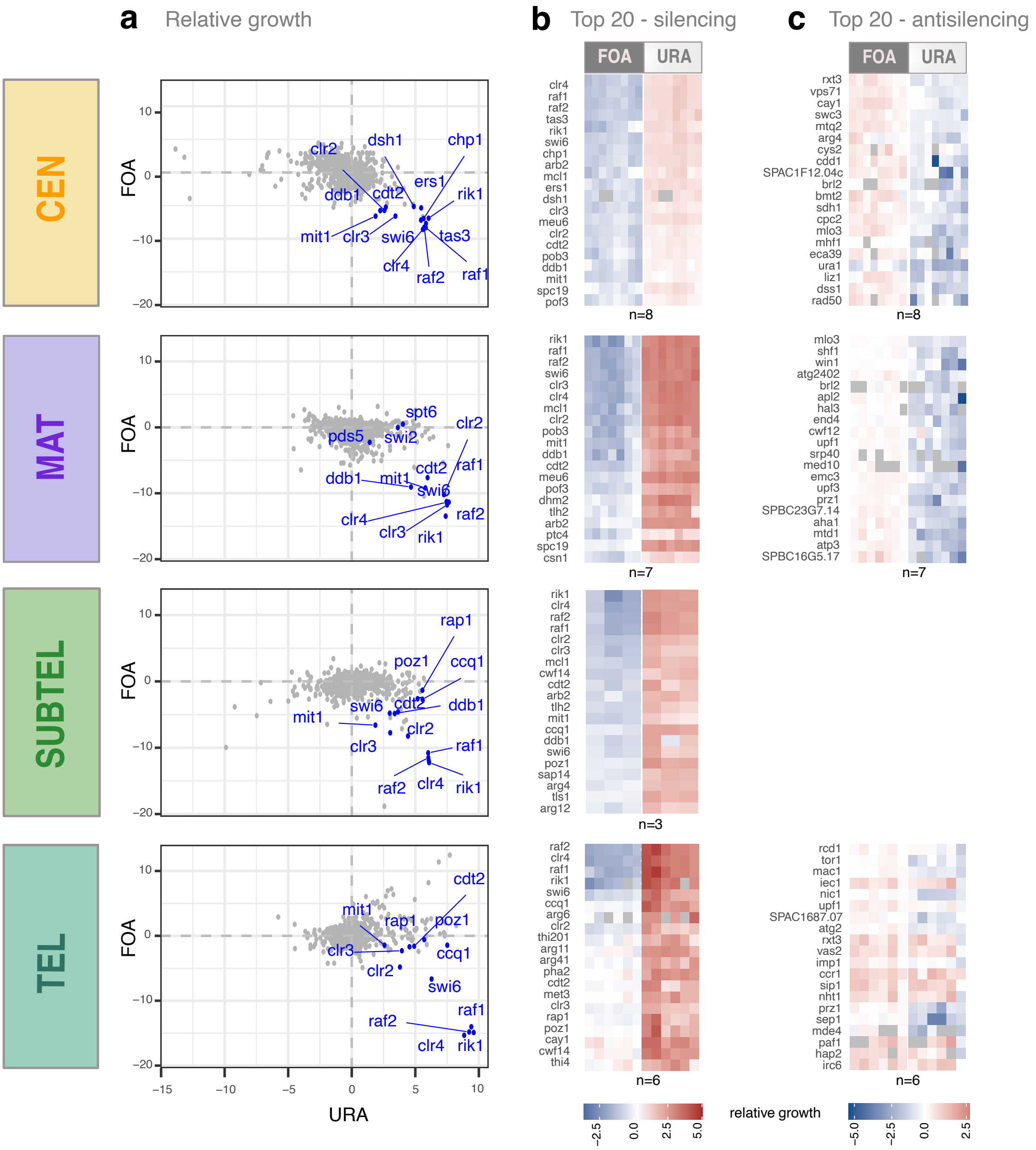

Figure S4. Top candidates among identified silencing and anti-silencing factors. a) Scatterplots display log_2-transformed relative growth values (URA, FOA) for each reporter screen. Median values of 3-8 independent biological replicates (*CEN*: 8; *MAT*: 7; *SUBTEL*: 3; *TEL*: 6; each with 2-4 technical replicates) are shown. Blue dots represent known silencing factors (refer to Suppl. Table S4) detected in the screens. **b-c)** Heatmaps show the relative growth values (log_2) of the top 20 silencing (b) and anti-silencing (c) candidates. Values are derived from independent biological replicates, with each biological replicate calculated as the average of 2-4 technical replicates.

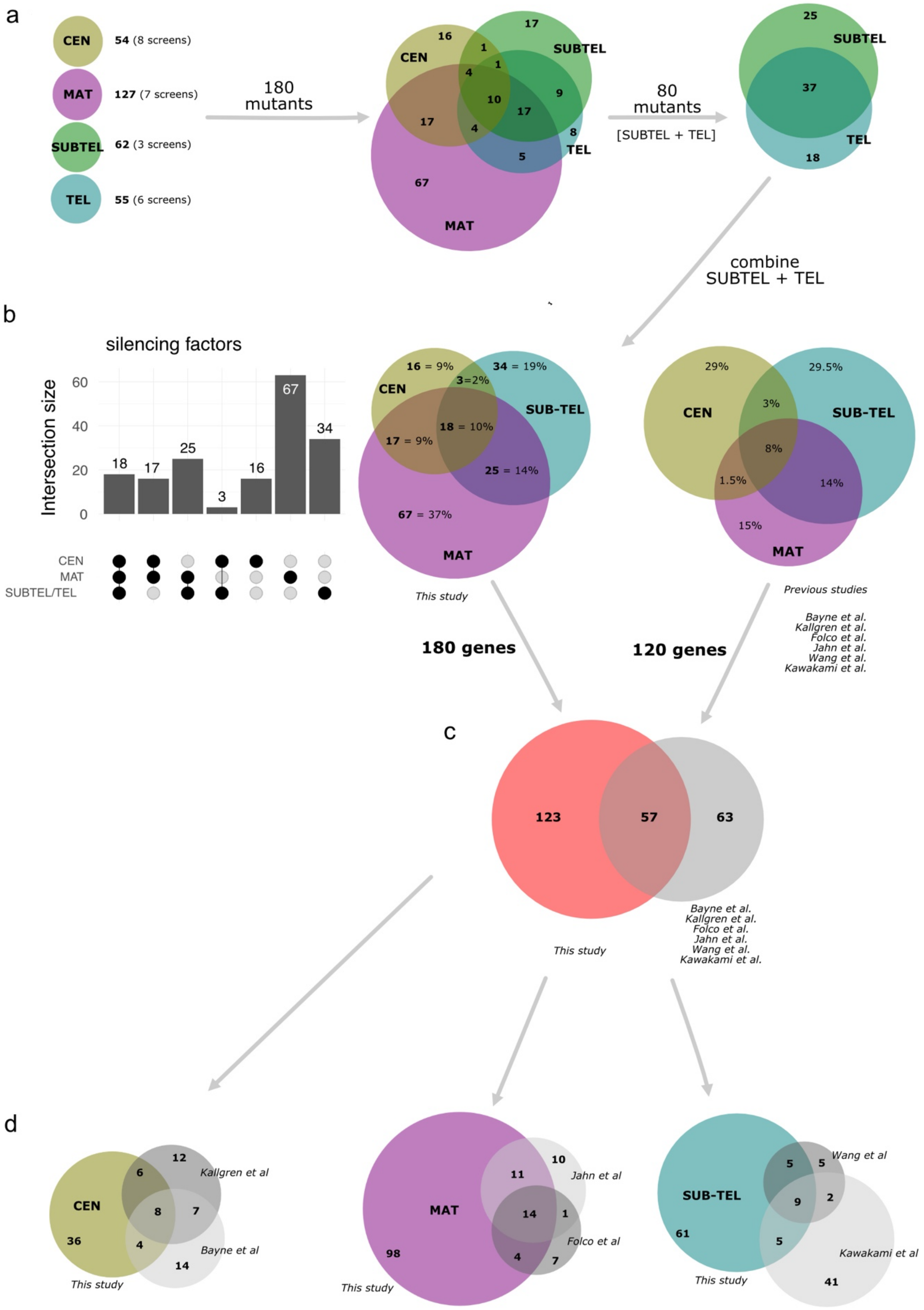

Figure S5. Comparative analysis of silencing hits from this study and previous genome-wide screens. a) Venn diagram illustrating the number of silencing hit candidates identified by this study, highlighting the shared defects detected across the four heterochromatic reporter screens. Notably, while overlap between individual reporter screens is limited, a significant overlap is observed between *SUBTEL* and *TEL* screens. **b)** Overlap of hits identified in this study, as shown by Upset plot (right) and Venn diagram (middle), in comparison to previous studies (right). Note, for clearer visualization, candidates from different studies identified by identical or similar (e.g., *SUBTEL, TEL*) reporter systems have been aggregated. **c)** Venn diagram showing the overlap of the total number of hits between this study and previous genome-wide screens. **d)** A series of Venn diagrams illustrate the overlap in domain-specific silencing hits identified in this study compared to those found in previous genome-wide screens.

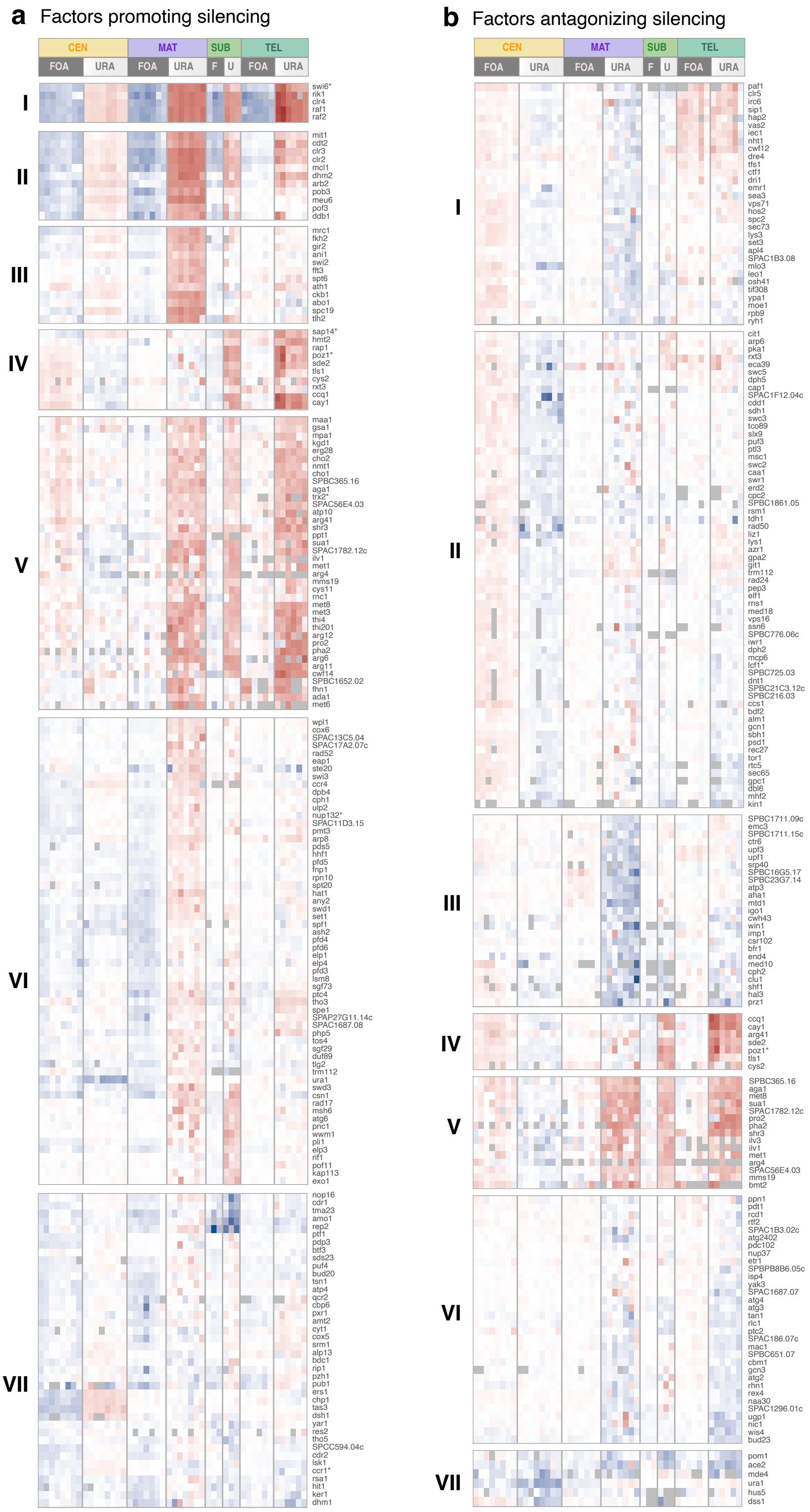

Figure S6. Cluster groups established by k-means clustering of factors promoting or antagonizing silencing. Heatmaps display relative growth values of mutants exhibiting **(a)** significantly reduced and **(b)** significantly enhanced silencing. In this analysis, 176 silencing and 179 anti-silencing mutants were examined. The data represent values derived from 3-8 independent biological replicates (*CEN*: 8; *MAT*: 7; *SUBTEL*: 3; *TEL*: 6; each with 2-4 technical replicates). The gene order within each cluster was determined through subsequent hierarchical clustering (see Methods). Note that 4 silencing mutants (out of 180) and 10 anti-silencing mutants (out of 189) were excluded from the k-means clustering due to missing values. In cases where mutants were mis-annotated in the gene deletion collection, the correct gene name is indicated by an asterisk (see also Suppl. Table S8 and Methods).

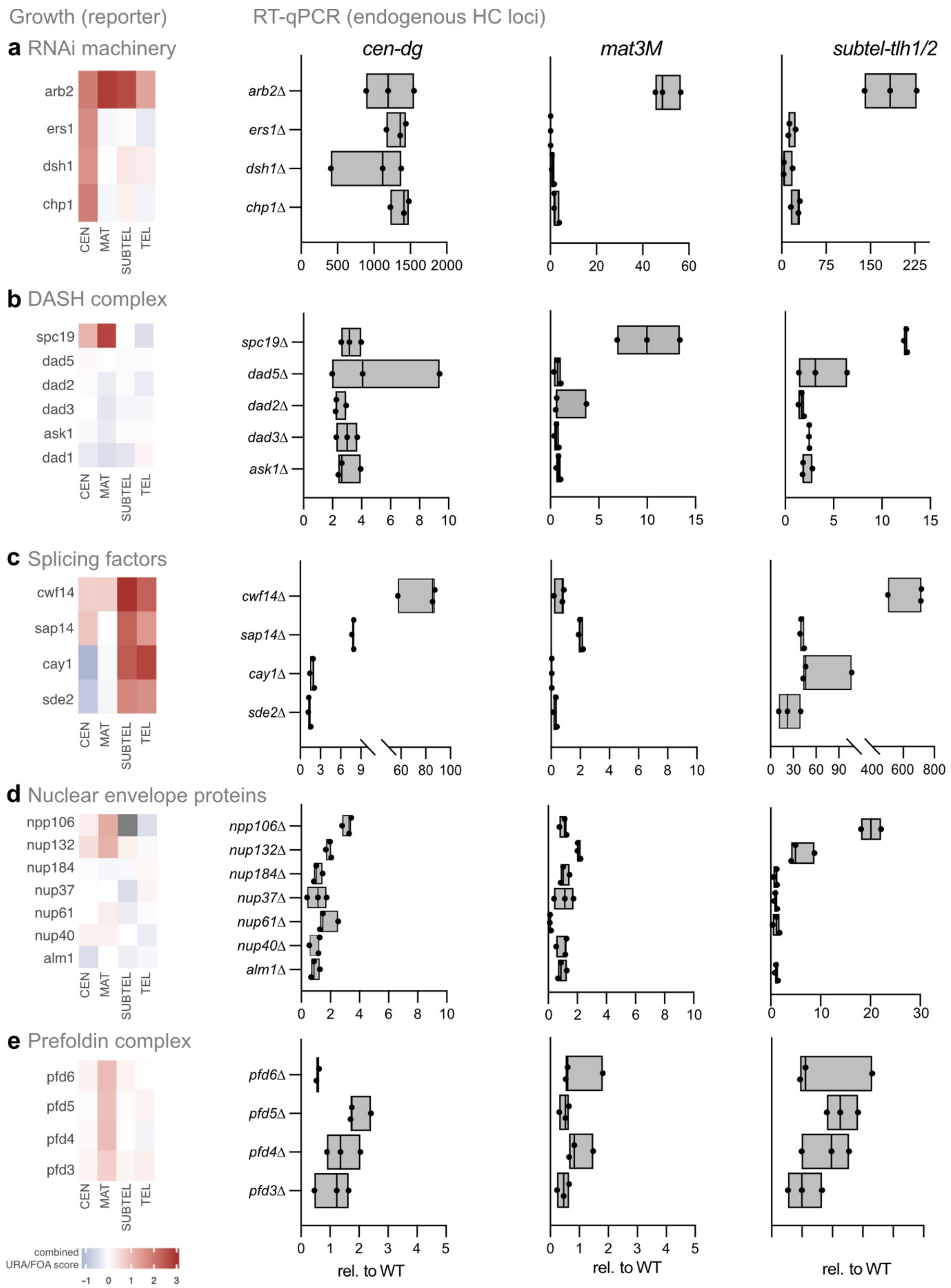

Figure S7. RT-qPCR analysis of mutants associated with specific protein complexes or functional pathways. Reporter growth-based heatmaps and expression analysis of endogenous heterochromatic transcripts in mutants associated with **(a)** RITS, **(b)** DASH, **(c)** splicing factors, **(d)** nuclear envelope protein, and **(e)** prefoldin complex. Heatmaps display median values of combined FOA/URA scores (log_2). The scores were calculated from 3-8 independent biological replicates (*CEN*: 8; *MAT*: 7; *SUBTEL*: 3; *TEL*: 6; each with 2-4 technical replicates). Plots accompanying each heatmap illustrate the endogenous transcript levels at three heterochromatic loci (*cen-dg, mat3M,* and *tlh1*), as determined by RT-qPCR. Transcript levels, normalized against *act1*, are presented relative to the WT mean values (n = 3 independent biological replicates).

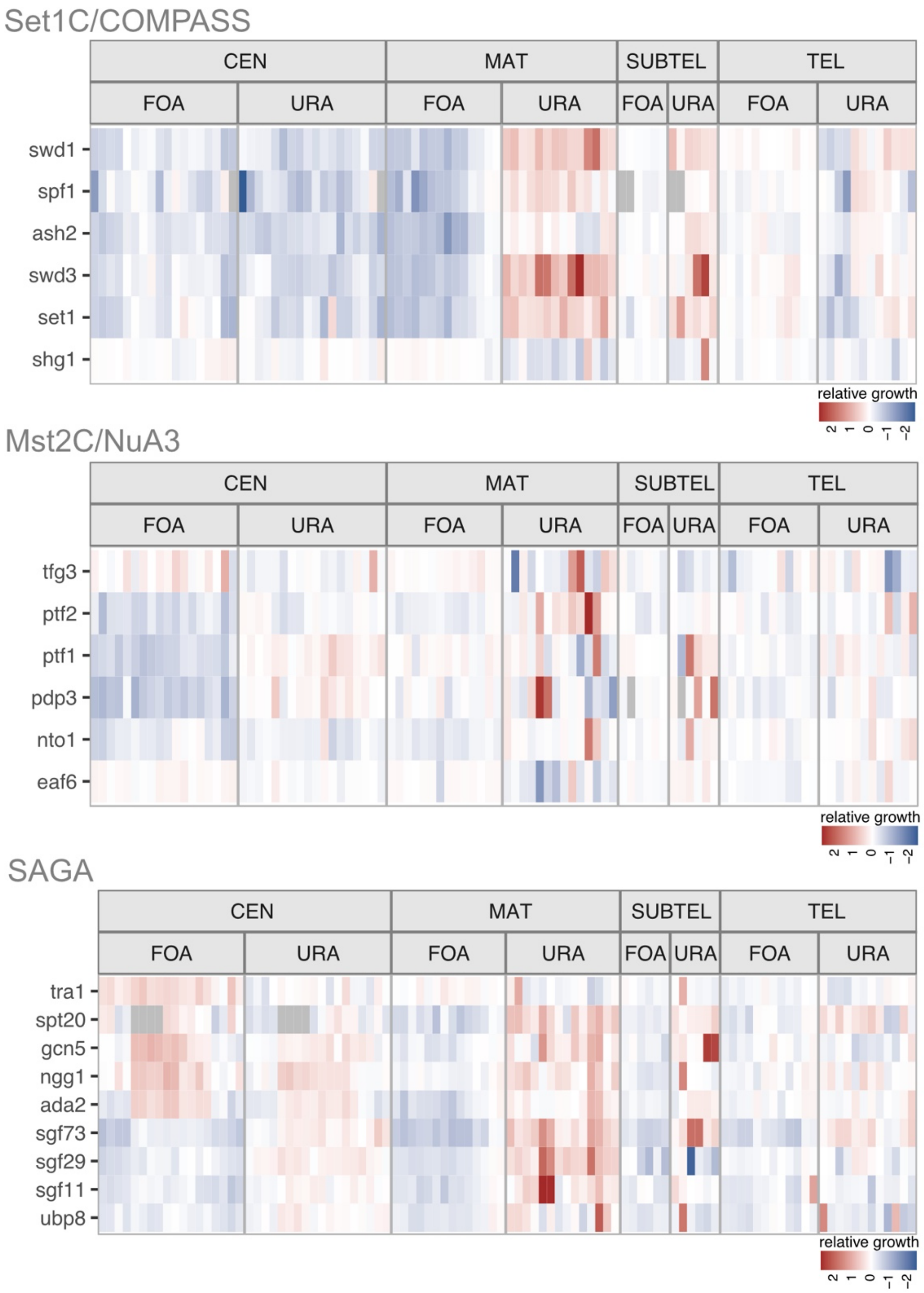

Figure S8. Silencing defects of mutants associated with known chromatin-organizing complexes. Heatmaps show reporter-based relative growth values (log_2) derived from the genome-wide silencing assays. Mutants associated with a) Set1C/COMPASS, b) Mst2C/NuA3, and c) SAGA complexes are shown.

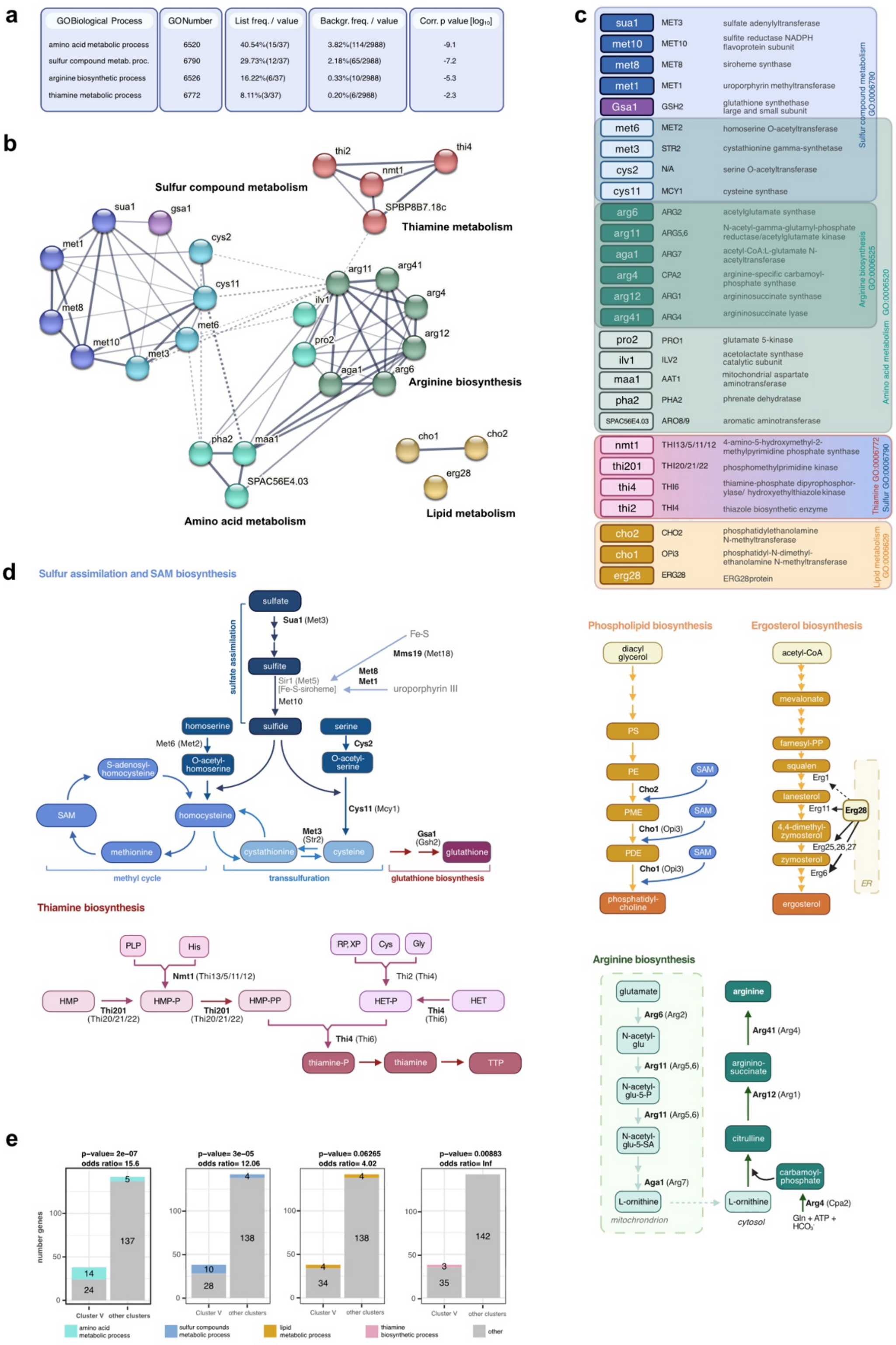

Figure S9. Gene ontology and interaction analysis of silencing factors involved in metabolic processes. a) Table presents selected results from gene list enrichment analysis of the 38 genes from silencing factor cluster V. This analysis used the AnGeLi web-based-tool (Bitton et al., 2015) and employed a two-tailed Fisher's exact test and a false-discovery rate of 0.05 was used for the analysis. **b**) STRING analysis (https://string-db.org/; (Szklarczyk et al., 2023) visualizes interactions among silencing factors from metabolic pathways detailed in (a). **c)** Table list *S. pombe* gene names, along with their *S. cerevisiae* homologs (when available), and provides details on molecular function, metabolic pathways, and associated GO terms. **d**) Schematics illustrating key metabolic pathways in sulfur assimilation/ SAM biosynthesis pathway, phospholipid biosynthesis, ergosterol biosynthesis, thiamine biosynthesis, and arginine biosynthesis. Proteins with bold names represent mutants showing significantly reduced silencing; protein names in brackets indicate *S. cerevisiae* homologs). **e)** Enrichment analysis of the genes involved in metabolic processes across different silencing factor clusters. Bar plots compare Cluster V to other clusters regarding the number of genes associated with distinct metabolic processes, with p-value and odds ratio indicating the significance and strength of association, as determined by Fisher's exact test. Tables and schemes in a), c), and d) created in BioRender. Braun, S. (2023) BioRender.com/e59u408 and BioRender.com/d83d446

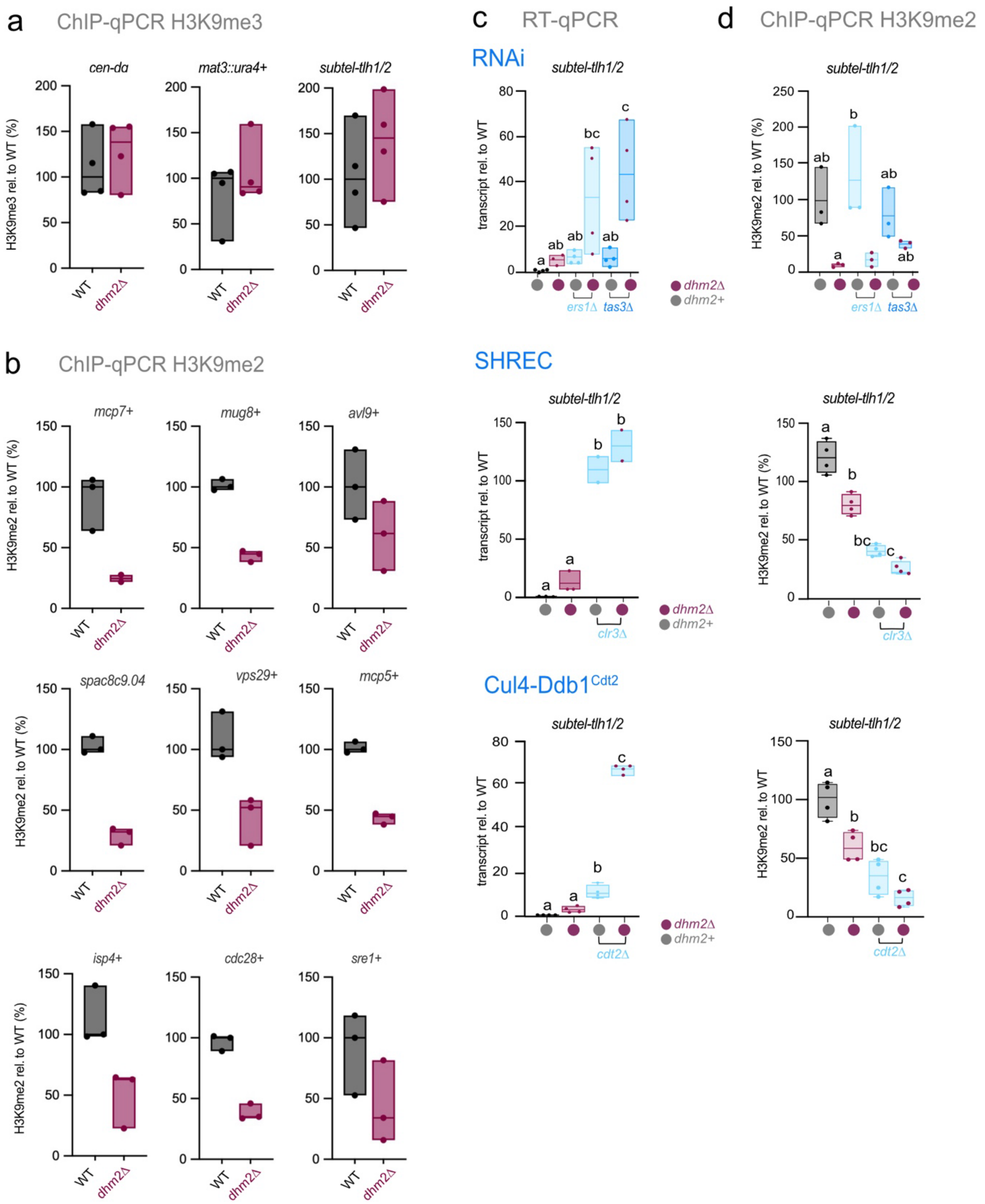

Figure S10. Heterochromatin structure and silencing at constitutive and facultative heterochromatin in *dhm2*Δ. a) ChIP-qPCR analysis of H3K9me3 enrichment at *cen-dg* repeats and the *mat3::ura4* reporter gene (*n* = 3-4 independent biological replicates). **b**) ChIP-qPCR analysis of H3K9me2 levels of facultative heterochromatin islands (*n* = 3 independent biological replicates). **c**) RT-qPCR quantification of *tlh1* transcript levels quantified by RT-qPCR in the indicated strains (*n* = 3-4 independent biological replicates). Data are normalized to *act1* transcript level and presented relative to WT median value. **d)** ChIP-qPCR analysis of H3K9me2 levels at $tlh1^+$ in the indicated strains (*n* = 3-4). For ChIP analysis in (a), (b), and (c), immunoprecipitated (IP) samples, normalized to input, are further standardized against the average of two euchromatic loci ($act1^+$ and $tef3^+$). Data are presented relative to the WT median value. Statistical analysis for (c) and (d) employed one-way ANOVA, with Tukey's *post hoc* test identifying significant differences at $P < 0.05$. Letters denote groups with significant differences.

Figure S11. Heterochromatin establishment and maintenance at endogenous and ectopic heterochromatin domains.

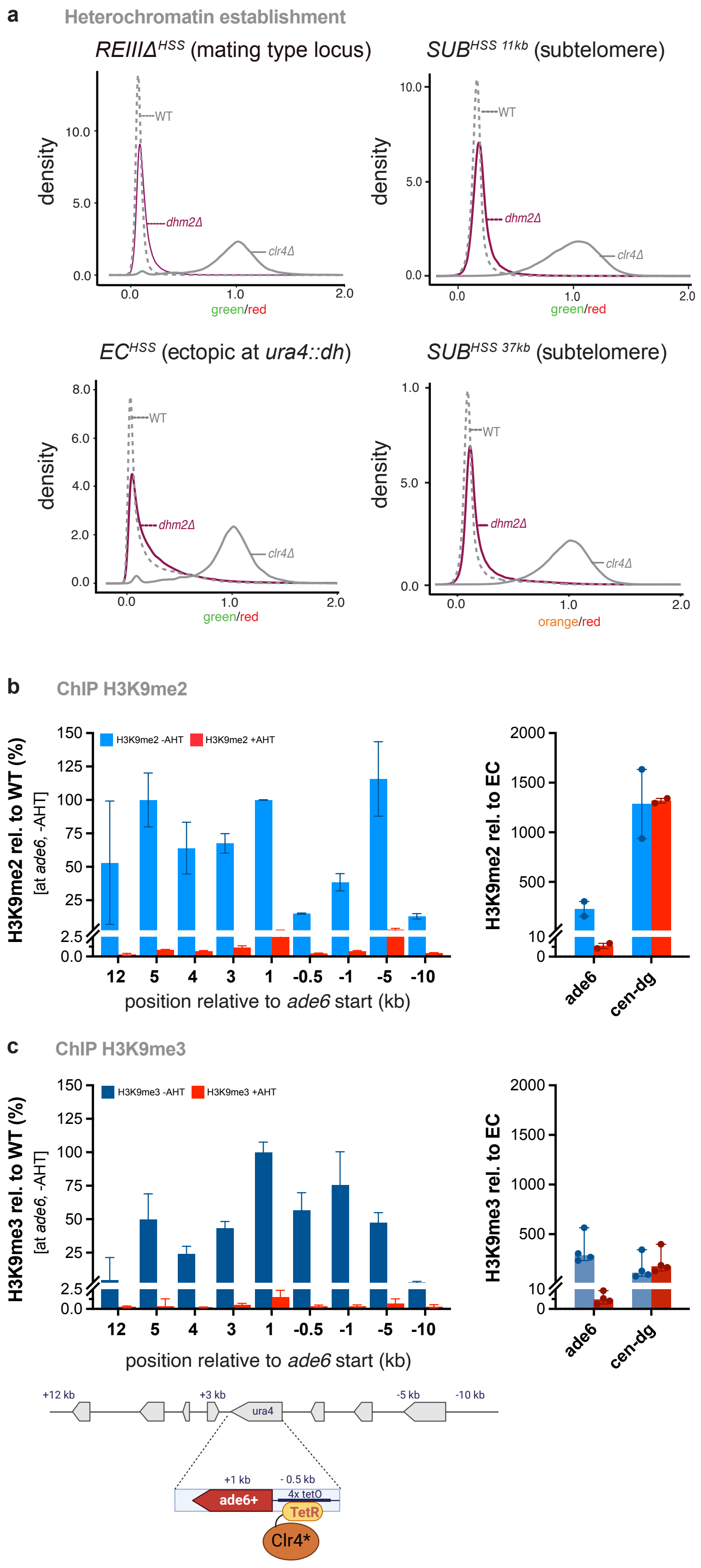

a) Assessment of heterochromatin establishment at single-cell level**.** Density plots of flow cytometry measurements illustrating reporter expression in wild-type (WT; dashed line), *dhm2Δ* (maroon solid line), and *clr4Δ* (gray solid line) strains across various heterochromatin domains. Top left: *cenH* region of the mating type locus (ΔREIII^{HSS}). Bottom lef: Ectopic locus created by inserting the pericentromeric *dh* element next to endogenous *ura4*$^+$ locus (*EC*^{HSS}). Top right: subtelomeric locus 11 kb downstream of the telomeric repeats (*SUB*$^{HSS\ 11kb}$). Bottom right: Subtelomeric locus 37 kb downstream of the telomeric repeats (*SUB*$^{HSS\ 37kb}$). The x-axis displays relative expression values of the SF-GFPsp (green) or the mKO2sp (orange) reporter. The y-axis represents the density of the cell population. Reporter expression is normalized to E2C (red; noise filter) expressed from an adjacent locus located in euchromatin and is shown relative to the median expression in the *clr4*Δ strain. **b)** and **c)** ChIP-qPCR analysis of H3K9me2 and H3K9me3 enrichment. Left panels show input-normalized ChIP-qPCR analysis of H3K9me2 and H3K9me3 enrichment at the *4xtetO-ade6* locus in WT cells. IP samples are normalized to the average of two euchromatic loci (*act1*$^+$ and *tef3*$^+$) and shown relative to enrichment at the *tetO* binding site in the absence of AHT (set to 100%). Median values from n = 2 and 4 independent experiments for H3K9me2 and H3K9me3, respectively, are shown with error bars representing the deviation from the median. Positions relative to the *4xtetO* nucleation site are indicated (in kb). Right panels compare H3K9me2/me3 enrichment at *ade6* locus (corresponding to the 1 kb position in the left panels) and the endogenous pericentromeric *dg* repeats (*cen-dg*). Data show IP samples relative to euchromatin (EC). Consistent with H3K9me2 being primarily associated with RNAi-controlled heterochromatic loci, H3K9me2 accumulates to higher levels at the *dg* repeats, while H3K9me3 shows comparable levels at *ade6* and *cen-deg*.

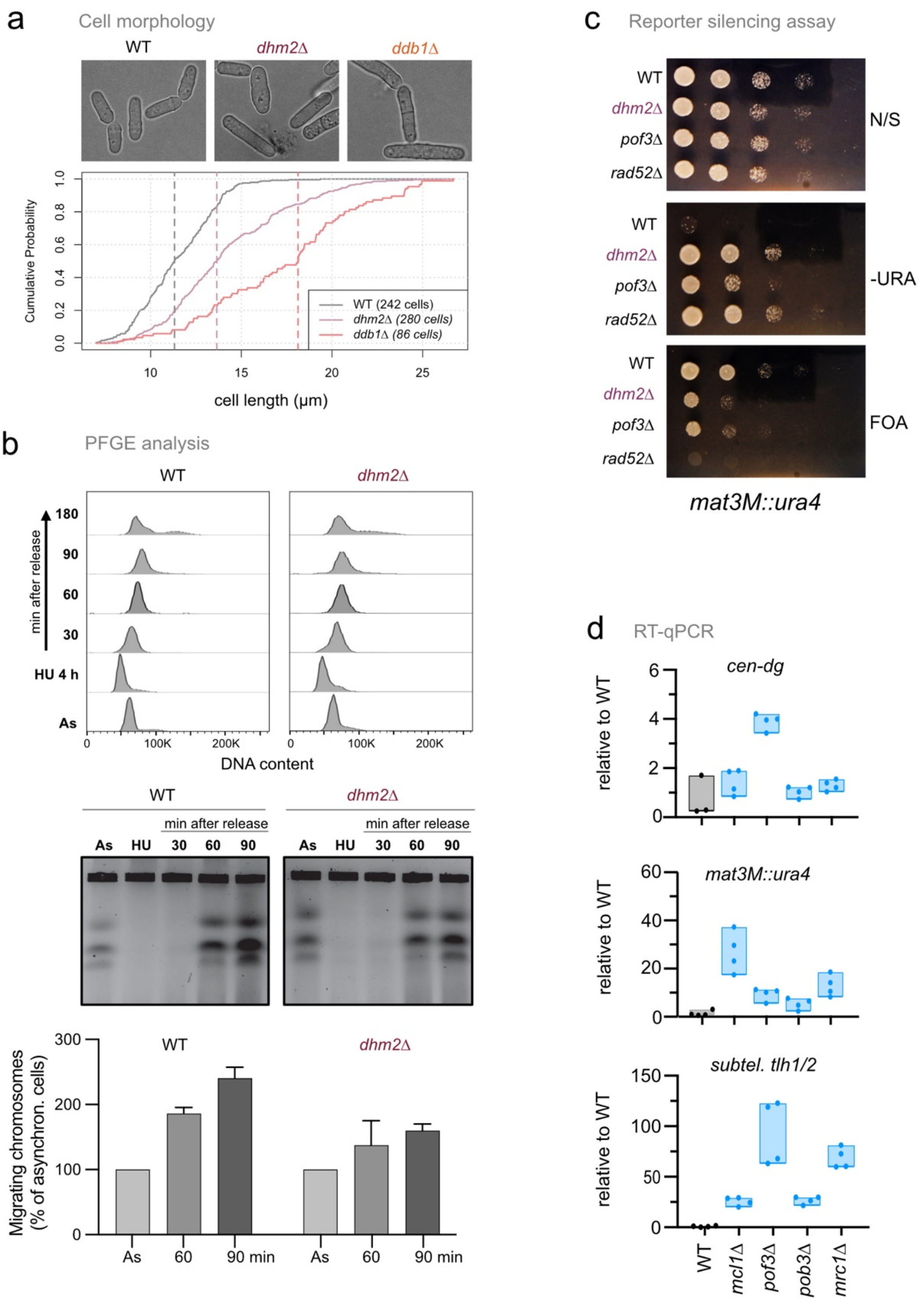

Figure S12. Cell cycle progression and heterochromatin silencing in *dhm2*Δ and mutants associated with DNA replication defects. a) Elongation phenotype of *dhm2*Δ. The length of non-dividing cells (absence of septum) of WT and *dhm2*Δ cells were measured. As a positive control, cells lacking the adaptor protein Ddb1 of the E3 ubiquitin ligase Cul4-Ddb1-Cdt2, which results in the accumulation of the ribonucleotide reductase inhibitor Spd1, were examined. Shown are representative images (top) and cumulative probability plots (bottom). **b)** Cell cycle progression. HU-arrested WT and *dhm2*Δ cells were released into HU-free media. Top panel: DNA content was measured by flow cytometry. Mid panel: Chromosome migration was analyzed by PFGE (pulse field gel electrophoresis). Bottom panel: Quantification of signal intensities compared to asynchronous cells (As; set to 100%). **c)** Reporter silencing assay. Tenfold serial dilutions of indicated strains were plated on EMM (minimal growth medium) under various conditions (N/S, non-selective; -URA, without uracil; FOA, supplemented with fluoroorotic acid) and incubated for 3 days at 32˚C. **d)** RT-qPCR quantification of heterochromatic transcripts (*cen-dg*, *mat3::ura4* and *tlh1/2*) in mutants lacking $Mcl1^{Ctf4}$, $Pof3^{F\text{-}box}$, $Pob3^{FACT}$ and $Mrc1^{Claspin}$. Data are normalized to *act1* transcript level and presented relative to the WT median value (n = 4). **c)** Viability efficiency (%) was determined by counting the number of complete tetrad (four-spored ascus) from the representative strains
